# Peptide‐Directed Binding for the Discovery of Modulators of α‐Helix‐Mediated Protein–Protein Interactions: Proof‐of‐Concept Studies with the Apoptosis Regulator Mcl‐1

**DOI:** 10.1002/anie.201705008

**Published:** 2017-07-25

**Authors:** Andrew Michael Beekman, Maria Anne O'Connell, Lesley Ann Howell

**Affiliations:** ^1^ School of Pharmacy University of East Anglia Norwich Research Park, Norwich Norfolk NR4 7TJ UK; ^2^ School of Biological and Chemical Sciences Queen Mary University of London Mile End Road London E1 4NS UK

**Keywords:** apoptosis, drug discovery, medicinal chemistry, protein–protein interactions, solid-phase synthesis

## Abstract

Targeting PPIs with small molecules can be challenging owing to large, hydrophobic binding surfaces. Herein, we describe a strategy that exploits selective α‐helical PPIs, transferring these characteristics to small molecules. The proof of concept is demonstrated with the apoptosis regulator Mcl‐1, commonly exploited by cancers to avoid cell death. Peptide‐directed binding uses few synthetic transformations, requires the production of a small number of compounds, and generates a high percentage of hits. In this example, about 50 % of the small molecules prepared showed an IC_50_ value of less than 100 μm, and approximately 25 % had IC_50_ values below 1 μm to Mcl‐1. Compounds show selectivity for Mcl‐1 over other anti‐apoptotic proteins, possess cytotoxicity to cancer cell lines, and induce hallmarks of apoptosis. This approach represents a novel and economic process for the rapid discovery of new α‐helical PPI modulators.

Protein–protein interactions (PPIs) regulate many processes in life, both in healthy and disease states,^1^ and almost two thirds of protein–protein interfaces have α‐helical binding motifs.^2^ However, targeting PPIs can be difficult owing to their large hydrophobic binding surfaces.^3^ There are currently three commonly employed approaches to develop modulators of PPIs:^4^ fragment screening,^5^ computational screening and drug design,^6^ and the exploration of peptides and peptidomimetics.^7^ However, computational design and fragment screening require large libraries of molecules and extensive synthetic work, often resulting in non‐selective compounds.^3^ Peptides are challenging drug leads because in vivo their efficacy can be compromised owing to a loss of secondary structure, poor cellular uptake, and susceptibility to proteolysis.^8^

The work described here exploits the advantages of the above approaches while limiting their weaknesses. This approach, termed peptide‐directed binding, utilizes the tight and selective binding of α‐helical peptides that govern PPIs as a framework for the discovery of small molecules. Sections of the natural peptide are employed to identify a small‐molecule fragment that emulates the peptide.

Inspiration for this approach was taken from the REPLACE strategy of McInnes and co‐workers^9^ and the chimeric inhibitors of the 14‐3‐3/Tau PPI developed by Ottmann and co‐workers.^10^ The work also exploits the advantages of altering peptidic binders, demonstrated by the groups of Gellman,^11^ Fairlie,^12^ and Wilson.^13^ The technique demonstrated here improves these strategies by substituting up to ten amino acids with one small‐molecule fragment and rapidly supplanting the entire peptide chain with a small‐molecule modulator.

In this proof‐of‐concept study, the α‐helical PPI of Mcl‐1 and Noxa, members of the apoptosis‐regulating Bcl‐2 family of proteins, was employed as an example.^14^ Anti‐apoptotic proteins (e.g., Bcl‐2, Bcl‐x_L_, and Mcl‐1) are often overexpressed in cancer, contributing to the development of the tumor and resistance to current therapies.^15^

Noxa displays high selectivity towards Mcl‐1.^16^ The NoxaB (NoxaB‐(75–93)‐C75A) peptide contains the 19 amino acids (AAQLRRIGDKVNLRQKLLN) from the BH3 binding region of Noxa, and has been shown to bind tightly as an α‐helix in the binding groove of Mcl‐1.^17^ NoxaB was divided into amino acids 75–84 (AAQLRRIGD) and 85–93 (KVNLRQKLLN); each section possesses two key binding residues (L78, I81, V85, and Q89),^17^ and reactive terminals were attached (Scheme 1 A). These peptides possessing reactive terminals displayed no appreciable binding affinity for Mcl‐1 (IC_50_>100 μm in a previously reported competitive fluorescence anisotropy (FA) assay),16c in accordance with results reported by Colman and co‐workers.^17^ These reactive terminals were then utilized to perform copper‐catalyzed azide–alkyne cycloaddition (CuAAC) reactions to attach small‐molecule fragments (R^1^ and R^2^).^18^ If binding affinity is restored for these small‐molecule/peptide hybrids (AAQLRRIGD−R^1^ and R^2^−KVNLRQKLLN), then the attached small‐molecule fragment may represent a good emulator for that particular section of NoxaB.

**Scheme 1:**
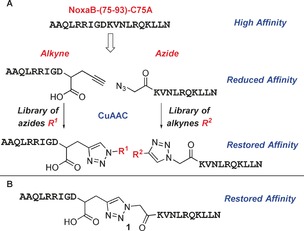
A) Concept of using peptide‐directed binding to target PPIs. B) Extended NoxaB peptide fragment that demonstrated restored affinity for Mcl‐1.

As an initial control for this strategy, the two peptide fragments were clicked together to generate an extended NoxaB peptide, which had an IC_50_ value of 7.23±0.88 μm, compared to 650±130 nm for NoxaB, highlighting the suitability of the approach (Scheme 1 B).

To economize peptide‐directed binding, covalent docking studies using the Schrodinger Suite were employed to assist in the identification of the small‐molecule fragments most likely to mimic a section of the NoxaB peptide. The crystal structure geometry of the NoxaB peptide with Mcl‐1 (PDB No. 2NLA)^17^ was modified; virtually, amino acids 85–93 of NoxaB were removed, and propargylglycine was attached to the C‐terminus of amino acids 75–84. Covalent docking studies were performed on a catalogue of azides, modelling a Huisgen cycloaddition (see the Supporting Information for details). The results were scored and ranked,^19^ and those highly ranked structures that were synthetically and economically viable were chosen for synthesis. Similarly, amino acids 75–84 of the peptide were virtually removed, and azidoacetic acid was attached to the N‐t*erm*inus of amino acids 85–93.

In this manner, we selected sixty hybrid compounds, which were synthesized and screened for their ability to disrupt the Mcl‐1/Noxa PPI. The peptide consisting of amino acids 85–93 (KVNLRQKLLN) of NoxaB was prepared by solid‐phase peptide synthesis (SPPS), and azidoacetic acid was used to cap the peptide (Scheme 2, middle). Subsequently, various alkynes were exposed to the peptide on the resin in the presence of Cu(MeCN)_4_PF_6_ and DIPEA in DMF to achieve CuAAC reactions. The use of an N‐coordinated Cu^I^ source was found to be advantageous, as other Cu^I^ sources gave lower yields, presumably owing to sequestration of the copper catalyst by peptidic coordination. Cleavage from the resin and reverse‐phase HPLC provided a library of 35 R^2^–KVNLRQKLLN hybrids. An analogous method was applied to prepare 25 AAQLRRIGD–R^1^ hybrids with a propargylglycine‐terminated SPPS resin (Scheme 2, top).

**Scheme 2:**
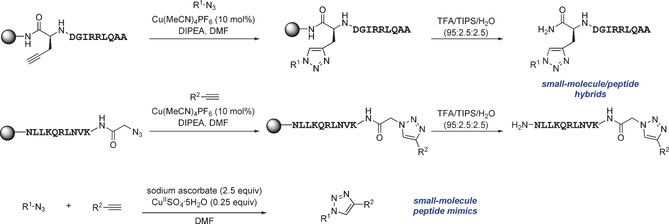
Synthesis of the small‐molecule/peptide hybrids by SPPS and subsequent synthesis of small‐molecule peptide mimics. DIPEA=diisopropylethylamine, DMF=dimethylformamide, TFA=trifluoroacetic acid, TIPS=triisopropylsilane.

The ability of these 60 hybrids to inhibit the interaction of Mcl‐1 and FITC‐Noxa was examined in an FA assay; 23 compounds (30 %) were identified as hits (defined as having an IC_50_<100 μm). Eight of these hits contained amino acids 75–84, and 13 hits were derived from amino acids 85–93 (Table 1; see also the Supporting Information, Table S1).

**Table 1:** IC_50_ values for inhibition of the binding of FITC‐NoxaB to Mcl‐1 of small‐molecule/peptide hybrids.^[a]^

	Peptide	Small molecule	FA IC_50_ [μm]			Peptide	Small molecule	FA IC_50_ [μm]
**2**	AAQLRRIGD	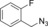	0.4±0.3		**9**	KVNLRQKLLN	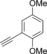	0.1±0.1
**3**		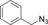	0.7±0.8		**10**		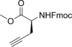	1.2±9.5
**4**		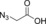	5.8±4.6		**11**		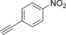	3.5±1.5
**5**		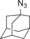	<100^[b]^		**12**		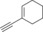	4.3±0.9
**6**		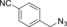	<100^[b]^		**13**		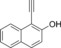	8.2±1.9
**7**		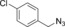	<100^[b]^		**14**		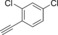	8.3±3.6
**8**		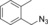	<100^[b]^		**15**		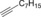	<100^[b]^

[a] IC_50_ values determined by non‐linear regression of at least three experiments. Errors are the transformed greater extreme of the standard error. [b] The hybrid compound displayed an IC_50_ value of <100 μm and >10 μm, accurate value not determined. Fmoc=9‐fluorenylmethylcarbonyl.

The orthogonal nature of the reaction enabled the combination of azide and alkyne small‐molecule fragments to generate a library of small‐molecule triazoles that in theory have an increased likelihood of possessing characteristics that emulate the entire NoxaB peptide. The identified hybrid molecules suggested 104 triazoles for preparation. We selected 35 for synthesis and evaluated them in an FA assay (Scheme 2, bottom). Nineteen (54 %) of the triazole compounds showed an IC_50_<100 μm, and ten (27 %) compounds displayed an IC_50_<1 μm (Table 2 and Table S2).

**Table 2:** IC_50_ values for the inhibition of the binding of FITC‐NoxaB to Mcl‐1 of small molecules and cell growth inhibition of representative compounds towards the pancreatic cancer cells lines MiaPaCa‐2, BxPC‐3, and AsPC‐1.^[a]^

Compound		FA IC_50_ [nm]	MiaPaCa‐2 [μm]	BxPC‐3 [μm]	AsPC‐1 [μm]
**16**	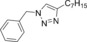	33±8	>100	>100	>100
**17**	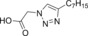	102±14	>100	>100	>100
**18**	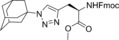	186±20	15.19±0.75	29.82±4.15	>100
**19**	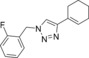	217±58	>100	21.60±8.83	>100
**20**	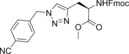	249±51	6.57±3.92	5.98±1.14	2.04±0.62
**21**	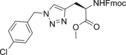	1260±142	1.85±3.17	2.81±0.40	>100
**22**	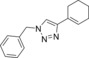	1680±694	>100	>100	>100
**23**	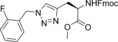	1700±230	19.52±1.35	25.21±2.66	10.54±1.53
**24**	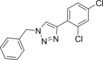	2210±877	2.14±0.56	10.66±2.72	2.28±1.44
**25**	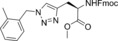	5500±3923	>100	>100	>100

[a] IC_50_ values determined by non‐linear regression of at least three experiments. Errors are the transformed greater extreme of the standard error.

The nature of this approach allows for structure–activity relationships (SARs) to be drawn without targeting specific modifications. The most potent compounds, **16** and **17**, both possess a heptyl chain, which provokes concerns about non‐specific hydrophobic events. However, not all of the synthesized compounds with the heptyl chain demonstrated activity (Table S2). Additionally, the Fmoc‐propargylglycine moiety proved effective in several of the identified binders (**18**, **20**, **21**, **23**, and **25**), but again was not a feature that was sufficient for binding on its own (Table S2). Concerns over the Fmoc protecting group were considered, but as these compounds are primarily meant as chemical probes or potential leads, further development may discover more effective alternatives. Indeed, the Fmoc group is somewhat reminiscent of structural features present in Souers’ A‐121047716d and Fesik's 2‐indole‐acylsulfonamides,^20^ which are highly potent and selective Mcl‐1 binders. Interestingly, the most potent small molecules did not result from the combination of the most potent small‐molecule peptide hybrids **2** and **9**, as might be expected. The combination of **2** and **9** demonstrated no appreciable ability to inhibit the interaction of Mcl‐1 and FITC‐Noxa. Additionally, the small‐molecule fragment of **9** did not appear in any small molecule that inhibited the Mcl‐1/FITC‐Noxa interaction. A possible explanation for this phenomenon is that some small‐molecule fragments, when bound to the peptide fragment, alter the helicity of the peptide, perhaps increasing the binding affinity of the peptide segment or altering the binding site.^21^ Further studies on the power of small‐molecule/peptide hybrids as PPI modulators are underway.

It has been shown that NoxaB is a selective Mcl‐1 binder.^17^ The deployment of the NoxaB peptide as a scaffold for discovering new compounds was envisaged to also impart this selectivity onto the new small‐molecule mimics. To examine this, we employed two in vitro FA assays with Bcl‐2 and Bcl‐x_L_, using an FITC‐tagged Bid peptide as our fluorescent marker.^22^ Navitoclax (ABT‐263) was employed as a positive control and as an additional indication that the FA assay was performing adequately.16b Excitingly, compounds **16**–**25** and an additional nine compounds (see the Supporting Information) displayed no appreciable binding to Bcl‐x_L_ or Bcl‐2 in our FA assays (Figure 1; see also the Supporting Information), demonstrating a minimum 20‐fold selectivity for Mcl‐1. To confirm that this result was not an artefact, all compounds were re‐examined in the Mcl‐1, Bcl‐2, and Bcl‐x_L_ assays twice more, with the NoxaB peptide and Navitoclax performing as expected. These results also provide some relief about the potential of non‐specific hydrophobic events caused by the heptyl chain or the Fmoc group.

**Figure 1:**
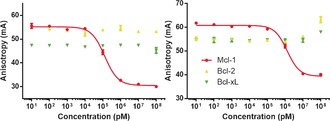
Representative titrations of compounds **18** (left) and **21** (right) on 5 nm FITC‐NoxaB peptide in the presence of 10 nm Mcl‐1 protein (red) or 5 nm FITC‐Bid peptide in the presence of 30 nm Bcl‐2 (yellow) and Bcl‐x_L_ (green), demonstrating no appreciable binding to Bcl‐2 and Bcl‐x_L_.

Compounds **16**–**25** were examined to determine if they displayed activity towards pancreatic cancer cells, which are known to overexpress members of the Bcl‐2 family, including Mcl‐1. An MTS assay was employed to examine the ability of the compounds to inhibit cell growth and affect metabolism, a potential indicator of cell death. Several compounds, notably **18**, **20**, **21**, **23**, and **24**, displayed activity towards the pancreatic cancer cell lines BxPC‐3, known to overexpress Mcl‐1, and MiaPaCa‐2, which overexpresses both Mcl‐1 and Bcl‐2. Interestingly, compound **19** only had activity against BxPC‐3 cells. The pancreatic cancer cell line AsPC‐1, which does not overexpress Mcl‐1, was also evaluated (Table 2). Two compounds, **18** and **21**, were ineffective against AsPC‐1 cells at the concentrations evaluated in our assay, which may suggest that they are acting through the inhibition of Mcl‐1. The difference in the magnitude of activity in cells compared to the in vitro FA assay is a commonly observed phenomenon, and largely due to cell permeability.16d, 23 Indeed, some of the more potent compounds in the FA assay showed no activity in the cellular assays, such as compound **17** (IC_50_=102±14 nm), suggesting an inability to cross the cell membrane. Additionally, subnanomolar binding affinities are often required for small molecules to compete with high‐affinity endogenous ligands.^24^ Compounds **18**, **20**, **21**, and **23** were highlighted by this assay, and selected for further examination.

To determine if these compounds are impacting on the intrinsic apoptosis pathway, as would be expected if they are binding to Mcl‐1 in cells,^25^ assays were performed that demonstrate induction of the apoptosis pathway. Compounds **18**, **20**, **21**, and **23** induced an increase in caspase‐3 activation in BxPC‐3 cells 4 h after treatment, as indicated by the cleavage of DEVD–pNa and a subsequent increase in optical density at 405 nm.^26^ Compounds **18** (25 μm, 0.053±0.02), **20** (5 μm, 0.036±0.009), **21** (5 μm, 0.046±0.004), and **23** (25 μm, 0.139±0.06) all induced an increase in optical density at 405 nm compared to a vehicle control (DMSO, 0.1 %, 0.003±0.005) in this assay (Figure 2 A). Additionally, compounds **18**, **20**, **21**, and **23** were shown to induce the externalization of phosphatidylserine on the cell surface,^26^ as demonstrated by the binding of annexin‐V‐FLUOS to BxPC‐3 cells, resulting in green fluorescence (Figure 2 B).

**Figure 2:**
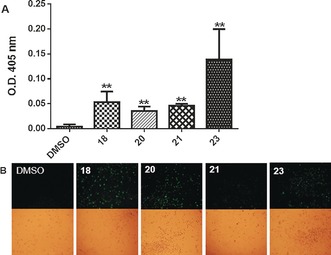
Representative small molecules induce hallmarks of apoptosis. A) Increase of caspase‐3 activation in BxPC‐3 cells 4 h after treatment. B) Externalization of phosphatidylserine, a hallmark of apoptosis, was evaluated by exposing BxPC‐3 cells to compounds **18** (25 μm), **20** (5 μm), **21** (5 μm), and **23** (25 μm) for 4 h followed by annexin‐V‐FLUOS. Images were taken at 10× magnification with a GFP filter (top) and white light (bottom).

In conclusion, by utilizing the selective NoxaB peptide as a framework, a library of novel Mcl‐1 binders that demonstrate selectivity for Mcl‐1 has been prepared. Peptide‐directed binding provided a high percentage of compounds with increased potency when compared to traditional methods of high‐throughput screening, by the application of a natural peptide framework and simple synthetic manipulations. Recent literature demonstrates that fragment‐based methods require the screening of approximately 15 000 fragments by NMR spectroscopy and extensive subsequent synthetic manipulations to generate a selective potent Mcl‐1 binder.^23^ Additionally, high‐throughput screening has been shown to have a hit rate of 0.2 % in recent studies targeting the Bcl‐2 family and other prominent cancer PPIs.^27^ The method exemplified here represents a significant economic improvement, in terms of both cost and time, when compared to both high‐throughput screening and fragment‐based methods and is a powerful new approach towards discovering modulators of α‐helical PPIs. A subset of the identified in vitro binders was found to possess activity towards cancer cell lines that overexpress Mcl‐1 and induce hallmarks of the apoptosis pathway. It is important to note that these compounds are currently unoptimized but still achieved low micromolar cellular activity, exemplifying the power of peptide‐directed binding to swiftly generate potential selective leads for challenging targets.

This proof‐of‐concept study has demonstrated that peptide‐directed binding is a technique that rapidly identifies new leads for α‐helical protein–protein interactions, as effectively exemplified for the Mcl‐1/Noxa PPI. Importantly, these compounds are able to mimic the selectivity of the natural scaffold. A recent review on Mcl‐1 inhibitors highlights that less than thirty compounds have been reported with comparable selectivity for Mcl‐1.^28^ Further studies are underway to structurally confirm the binding sites of the hybrid and small‐molecule compounds generated from peptide‐directed binding. It is expected that peptide‐directed binding is applicable to other α‐helical PPIs, such as the p53/hDM2 interaction, the Bcl‐x_L_/BIM interaction, or the HIV gp41 hexameric coiled‐coil fusion complex.^29^

## Conflict of interest

The authors declare no conflict of interest.

## Supporting information

As a service to our authors and readers, this journal provides supporting information supplied by the authors. Such materials are peer reviewed and may be re‐organized for online delivery, but are not copy‐edited or typeset. Technical support issues arising from supporting information (other than missing files) should be addressed to the authors.

Supplementary
