# Supplementary material for: Peptide‐Directed Binding for the Discovery of Modulators of α‐Helix‐Mediated Protein–Protein Interactions: Proof‐of‐Concept Studies with the Apoptosis Regulator Mcl‐1
Source: Angew Chem Int Ed Engl. 2017 Jul 25;56(35):10446–50. doi: 10.1002/anie.201705008 (PMC5577515; doi:10.1002/anie.201705008)
Supplement: Supplementary file 1 — Supplementary [file ANIE-56-10446-s001.pdf]

Angewandte Chemie
Eine Zeitschrift der Gesellschaft Deutscher Chemiker

Supporting Information

Peptide-Directed Binding for the Discovery of Modulators of α-Helix-Mediated Protein–Protein Interactions: Proof-of-Concept Studies with the Apoptosis Regulator Mcl-1

*Andrew Michael Beekman, Maria Anne O'Connell, and Lesley Ann Howell**

anie_201705008_sm_miscellaneous_information.pdf

Supporting Information

Contents

1. General Procedures 3
  Reagents and Solvents 3
  Physical Characterisation and Spectroscopic Techniques 3
  Chromatographic Techniques 3
2. Protein Expression 3
  Mcl-1 3
  Bcl-2 3
  Bcl-x_L 4
3. Synthesis 4
  FITC-NoxaB peptide 4
  NoxaB peptide 5
  FITC-Bid peptide 5
  Azido-peptide 5
  Alkynyl-peptide 5
  General azide formation 5
  General alkyne formation 5
  General Click reaction to peptides on resin 6
  General Click reaction of small molecules 7
  Experimental Data 7
4. Fluorescence Anisotropy Binding Assay 9
  Mcl-1 10
  Bcl-2 11
  Bcl-x_L 12
5. Molecular Docking 13
6. Cell Culture 14
7. Cellular assay 14
  MTS Cytotoxicity Assay 14
  Annexin-V/ PI staining 14
  Caspase 3 activation assay 15
8. NMR Spectra 16
9. Mcl-1 Fluorescence Anisotropy Assay 33
10. Bcl-2 and Bcl-xL selectivity Fluorescence Anisotropy Assay 37
11. Cell growth inhibition assay 41
12. References 43

1. General Procedures

Reagents and Solvents

All chemicals were reagent grade and were purchased from Sigma Aldrich, Fisher Scientific and Tokyo Chemical Industry. Fmoc-amino acids and coupling reagents were purchased from Novabiochem or AGTC Bioproducts. Anhydrous solvents were bought from Sigma Aldrich and assumed to conform to specification.

Physical Characterisation and Spectroscopic Techniques

^{1}H- and ^{13}C-NMR spectra were recorded on a Bruker spectrometer operating at 400 MHz (^{1}H) or 100 MHz (^{13}C) using the specified deuterated solvent. The chemical shifts for both ^{1}H- and ^{13}C- were recorded in ppm and were referenced to the residual solvent peak of $CHCl_3$ at 7.26 ppm (^{1}H) and 77.0 ppm (^{13}C). Multiplicities in the NMR spectra are described as s = singlet, d = doublet, t = triplet, q = quartet, m = multiplet, br = broad, and combinations thereof; coupling constants are reported in Hz. Assignments, where conspicuous, have been confirmed by appropriate 2D NMR experiments. MALDI was performed on Kratos Analytical Axima MALDI-TOF. Low resolution mass spectra were recorded using a Shimadzu LCMS 2010EV operated under electrospray ionisation in positive (ES+) mode. Accurate mass spectra were recorded at the EPSRC National Mass Spectroscopy Service Centre, Swansea. Melting points were recorded using open capillary tubes on a Mel-Temp electrothermal melting point apparatus, melting points are uncorrected. Infra-red spectra were recorded using a PerkinElmer Spectrum BX with ATR attachment.

Chromatographic Techniques

Analytical RP-HPLC was performed on an Agilent 1200 using an Agilent eclipse XDB-C18 column, 4.6 x 150mm, 5µM and a flow rate of 1 mL/min. Solvent A = Water + 0.05% TFA and Solvent B = MeOH + 0.05% TFA. Gradient 5% B → 95% B over 20 minutes. Detection wavelength 214 nm and 254 nm.

Semi-preparative RP-HPLC was performed on an Agilent 1200 using an Agilent eclipse XDB-C18 column, 9.4 x 250mm, 5µM and a flow rate of 4 mL/min. Solvent A = Water + 0.05% TFA and Solvent B = MeOH + 0.05% TFA. Gradient 5% B → 95% B over 20 minutes. Detection wavelength 214 nm and 254 nm.

2. Protein Expression

Mcl-1

The Mcl-1 protein used in this study is the same construction as the Mcl-1 described by Yu and Wang,[1] amino acid residues 152-189 of mouse Mcl-1 fused with amino acid residues 209-327 of human Mcl-1. The plasmid was generously provided by Yu and Wang. The protein with an N-terminal 8 x His tag was expressed in *E. coli* BL21(DE3)pLysS cells. Cells were grown at 37 °C in LB medium containing 100 µg/mL ampicillin and 34 µg/mL chloramphenicol to an OD_{600} value at 0.6. Protein expression was induced by 0.4 mM IPTG at 30 °C for 4 h. Cells were lysed in 25 mM Tris-HCl, pH 8.0 buffer containing 300 mM NaCl, 5 mM βME and 0.1 mg/mL PMSF. His-TEV-Mcl-1 protein was purified from the soluble fraction using Ni-NTA resin (QIAGEN), following the manufacturer's instructions.

Bcl-2

The Bcl-2 protein used in our study has the same construction as that used in the work of Fesik *et al.*[2] (Bcl-2/Bcl-x_L, isoform 2) which is composed of amino acid residues 1-34 of human Bcl-2, amino acid residues 29-44 of human Bcl-x_L, and amino acid residues 92-207 of human Bcl-2, resulting in good solubility in water and maintaining the biological function of human Bcl-2.

GPLGSEFMAHAGRTGYDNREIVMKYIHYKLSQRGYEWDAGDDVEENRTEAPEGTESEV
VHLTLRQAGDDFSRRYRRDFAEMSSQLHLTPFTARGRFATVVEELFRDGVNWGRIVAFF
EFGGVMCVESVNREMSPLVDNIALWMTEYLNRHLHTWIQDNGGWDAFVELYGPSMR.

Protein was purchased from Dundee Cell Product Ltd.

Bcl-x_L

The Bcl-x_L protein used in our study is a truncated construction of the full-length protein, with deletion of residues 45-84 on a large loop region and residues 210-233 at the C-terminal hydrophobic region, maintaining the biological function of Bcl-x_L.

GPLGSEFMSQSNRELVVDFLSYKLSQKGYSWSQFSDVEENRTEAPEGTESEAVKQALREA
GDEFELRYRRAFSDLTSQLHITPGTAYQSFEQVVNELFRDGVNWGRIVAFFSFGGALCVE
SVDKEMQVLVSRIAAWMATYLNDHLEPWIQENGGWDTFVELYGNNAAAESRKGQER.

Protein was purchased from Dundee Cell Product Ltd.

3. Synthesis

FITC-NoxaB peptide

The nineteen amino acid mouse NoxaB peptide AAQLRRIGDKVNLRQKLLN was synthesised on Rink amide MBHA resin (resin loading 0.6 mmol/g) using an automated peptide synthesiser. 100 mg of Rink Amide MBHA resin (0.06 mmol) was suspended in DMF (2 mL) and was allowed to swell for 20 minutes. The DMF was drained from the peptide vessel and Fmoc deprotection was carried out by addition of 2 x 2 mL of 40% piperidine in DMF, which was vortexed for 10 minutes. This was removed and the resin was washed with DMF (3 x 2 mL). The resin was then treated with a solution of Fmoc-Asn-Trt (4 equiv. compared to resin loading), to which HBTU (3.9 equiv.) and HOBt (4 equiv.) and DIPEA (8 equiv.) in DMF were added. The mixture was then vortexed for 30 min. The vessel was drained and the resin washed with DMF (3 x 2 mL). The coupling reaction was then repeated followed by Fmoc deprotection (2 x 2 mL 40% piperidine in DMF, 10 min) and finally the resin was washed with DMF. Subsequent amino acids were coupled in an identical fashion. After the final amino acid coupling reaction (alanine) and Fmoc deprotection, the resin was treated with Fmoc-aminohexanoic acid (Ahx, 4 equiv.), HBTU (3.9 equiv.), HOBt (4 equiv.) and DIPEA (8 equiv.) and reacted for 45 minutes. Subsequent Fmoc deprotection was followed by coupling with FITC (1.5 equiv.) and DIPEA (2 equiv.) in DMF overnight. The resin was washed with DCM (x 3) and 1:1 MeOH:DCM (x 3) to remove any residual DMF. The peptide was cleaved from the resin using 95:2.5:2.5 TFA:TIPS:H_2O (5 mL) and shaken for 3 h after which the cleavage cocktail was drained into a round bottom flask. The resin was washed with TFA (x 3) and the solutions combined and concentrated in vacuo. The peptide was precipitated using cold diethyl ether and filtered. The peptide was purified using automated reversed phase flash chromatography and lyophilised from water to yield a yellow solid. This was subsequently analysed using RP-HPLC (RT = 13.5 min) and MALDI (m/z 2707.34 M+H observed).

NoxaB peptide

The NoxaB peptide AAQLRRIGDKVNLRQKLLN synthesis was performed as described for FITC-NoxaB but the peptide was acetylated at the N-terminus after the addition of the final amino acid (alanine) using acetyl chloride (4 equiv.) and DIPEA (4 equiv.) in DMF and shaken for 45 minutes. The resin was washed with DMF (x 3), DCM (x 3) and 1:1 MeOH:DCM (x 3) to remove any residual DMF. The peptide was cleaved from the resin using 95:2.5:2.5 TFA:TIPS:H_2O (5 mL) and shaken for 3 h after which the cleavage cocktail was drained into a round bottom flask. The resin was washed with TFA (x 3) and the solutions combined and concentrated in vacuo. The peptide was precipitated using cold diethyl ether and filtered. The peptide was purified using automated reversed phase flash chromatography and lyophilised from water to yield a yellow solid. This was subsequently analysed using RP-HPLC (RT = 12.8 min) and MALDI (m/z 2248.85 M+H observed).

FITC-Bid peptide

The FITC-Bid peptide FITC-Ahx-EDIIRNIARHLAQVGDSMDR[3] synthesis was performed as described for FITC-NoxaB peptide, with the following alterations. The pseudo-proline amino acid dipeptide Fmoc-Asp(OtBu)-Ser(psiMe,Mepro)-OH (Novabiochem) to replace the DS sequence, in order to avoid aspartimide formation. Cleavage was performed using 90:2.5:2.5:2.5:2.5 TFA:TIPS:H_2O:EDT:TMSBr. This was subsequently analysed using RP-HPLC (RT = 16.7 min) and MALDI (m/z 2810.31 M+H observed).

Azido-peptide

The azido-peptide N_3Ac-KVNLRQKLLN was prepared as described for FITC-NoxaB with the following alterations. Following the final amino acid coupling (lysine) and Fmoc-deprotection, the resin was treated azidoacetic acid (N_3Ac, 4equiv.), HBTU (3.9 equiv.), HOBt (4 equiv.) and DIPEA (8 equiv.) and reacted for 45 minutes. To confirm synthesis a few beads were cleaved and purified as described for FITC-NoxaB. This was subsequently analysed using RP-HPLC (RT = 19.8 min) and MALDI (m/z 1307.82 M+H observed).

Alkynyl-peptide

The alkynyl-peptide Ac-AAQLRRIGD-($CH_2C{\equiv}C$)G was prepared as described for NoxaB with the following alterations. The amino acid bound to resin was Fmoc-L-propargylglycine. Subsequent deprotection, coupling, capping was performed as described for NoxaB. To confirm synthesis a few beads were cleaved and purified as described for FITC-NoxaB. This was subsequently analysed using RP-HPLC (RT = 19.9 min) and MALDI (m/z 1135.69 M+H observed).

General azide formation

Azides were either purchased directly from the supplier or prepared from the corresponding alkyl halides. Alkyl halide (e.g. 4-methoxybenzylbromide, 1 equiv.) and sodium azide (1.3 equiv.) were stirred in DMF (1 mL/mmol) at 50 °C for 4 hours. After cooling to room temperature water was added and the solution was extracted with diethyl ether. The organic layer was washed with water exhaustively, dried with $MgSO_4$, filtered and concentrated. The obtained azide, confirmed by IR spectroscopy, was used directly in the following step without further purification.

General alkyne formation

Alkynes were either purchased directly from the supplier or prepared from the corresponding aldehyde. Aldehyde (e.g. 2-fluorobenzaldehyde, 1 equiv.), methanol (1 mL/mmoml) and potassium carbonate (5 equiv.) were stirred at room temperature for 20 mins. The Bestmann-Ohira reagent[4] (1.3 equiv.) was added dropwise and the solution allowed to stir for 4 hours, at which time water was added. The solution was extracted with diethyl ether and the organic layer was washed with water and brine. The organic layer was dried with $MgSO_4$, filtered and concentrated. The obtained alkyne, confirmed by ^{1}H NMR spectroscopy, was used directly in the following step without further purification.

General Click reaction to peptides on resin

Peptide-resin complex (1 equiv.), alkyne (or azide, 1.1 equiv), $Cu(MeCN)_4PF_6$ (0.1 equiv.), DIPEA (2 equiv.) and DMF (0.5 mL/mmol) were shaken together for 16 hours. Bead were washed with DMF and MeOH. The product was cleaved and purified as described for FITC-NoxaB.

Table S1. Binding inhibition of FITC-NoxaB to Mcl-1 of small molecule-peptide hybrid compounds which were not pursued further in this study.

	Peptide	Small Molecule	FA IC_{50} (µM)		Peptide	Small Molecule	FA IC_{50} (µM)
**S1**	AAQLRRIGD	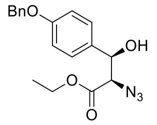	<100*	**S5**	KVNLRQKLLN	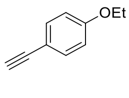	<100*
**S2**	KVNLRQKLLN	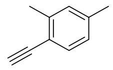	<100*	**S6**		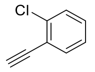	<100*
**S3**		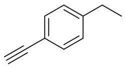	<100*	**S7**		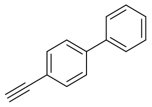	<100*
**S4**		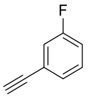	<100*				

*Hybrid compound demonstrated binding inhibition less than 100 µM and greater than 10 µM, accurate IC_{50} not determined.

1 analysed using RP-HPLC (RT = 18.1 min) and MALDI (m/z 2436.42 M+H observed).
2 analysed using RP-HPLC (RT = 14.3 min) and MALDI (m/z 1288.05 M+H observed).
3 analysed using RP-HPLC (RT = 15.5 min) and MALDI (m/z 1268.71 M+H observed).
4 analysed using RP-HPLC (RT = 14.0 min) and MALDI (m/z 1236.79 M+H observed).
5 analysed using RP-HPLC (RT = 10.2 min) and MALDI (m/z 1313.98 M+H observed).
6 analysed using RP-HPLC (RT = 15.2 min) and MALDI (m/z 1293.79 M+H observed).
7 analysed using RP-HPLC (RT = 14.9 min) and MALDI (m/z 1302.78 M+H observed).
8 analysed using RP-HPLC (RT = 15.7 min) and MALDI (m/z 1282.90 M+H observed).
9 analysed using RP-HPLC (RT = 14.8 min) and MALDI (m/z 1469.45 M+H observed).
10 analysed using RP-HPLC (RT = 15.7 min) and MALDI (m/z 1657.22 M+H observed).
11 analysed using RP-HPLC (RT = 13.2 min) and MALDI (m/z 1455.56 M+H observed).
12 analysed using RP-HPLC (RT = 12.7 min) and MALDI (m/z 1413.03 M+H observed).
13 analysed using RP-HPLC (RT = 16.2 min) and MALDI (m/z 1475.86 M+H observed).
14 analysed using RP-HPLC (RT = 14.3 min) and MALDI (m/z 1477.82 M+H observed).
15 analysed using RP-HPLC (RT = 16.1 min) and MALDI (m/z 1433.85 M+H observed).
S1 analysed using RP-HPLC (RT = 16.8 min) and MALDI (m/z 1476.30M+H observed).

S2 analysed using RP-HPLC (RT = 14.7 min) and MALDI (m/z 1437.62 M+H observed).
S3 analysed using RP-HPLC (RT = 14.1 min) and MALDI (m/z 1437.69 M+H observed).
S4 analysed using RP-HPLC (RT = 11.4 min) and MALDI (m/z 1429.48 M+H observed).
S5 analysed using RP-HPLC (RT = 13.5 min) and MALDI (m/z 1452.89 M+H observed).
S6 analysed using RP-HPLC (RT = 15.2 min) and MALDI (m/z 1445.97 M+H observed).
S7 analysed using RP-HPLC (RT = 15.7 min) and MALDI (m/z 1485.95 M+H observed).

General Click reaction of small molecules

Azide (1 equiv.), alkyne (1 equiv.), sodium ascorbate (2.5 eq), $CuSO_4.5H_2O$ (0.25 eq) and DMF (1 mL/0.5 mmol) were stirred together for 4 hours. The mixture was diluted with distilled water and extracted using diethyl ether. The organic layers were collected, washed exhaustively with water, dried with $MgSO_4$, and evaporated under reduced pressure. Purification was achieved with semi-preparative RP-HPLC prior to testing.

Experimental Data

16 *1-benzyl-4-heptyl-1H-1,2,3-triazole.*

M.P. 69 °C. ^{1}H NMR ($CDCl_3$); δ 7.30-7.15 (5H, m, Ph), 7.10 (1H, s, 5-H), 5.40 (2H, s, 1-CH_2), 2.59 (2H, t, ^3J = 7.8, 1′-CH_2), 1.54 (2H, m, 2′-CH_2), 1.20-1.10 (8H, m, 3′-6′-CH_2), 0.77 (3H, t, ^3J = 6.8, 7′-CH_3). ^{13}C NMR ($CDCl_3$); δ 149.2, 134.9, 129.0, 128.5, 127.9, 120.6, 53.9, 31.7, 29.3, 29.1, 28.9, 25.7, 22.6, 14.0. IR (neat): ν = 3113, 3063, 2917, 2850, 2362, 1211, 1052. LRMS (APCI): 258 (100, $[M+H]^+$). HRMS (ESI): $C_{16}H_{24}N_3$ calcd. $[M+H]^+$ = 258.1965, found: 258.1963.

18 *methyl (S)-2-((((9H-fluoren-9-yl)methoxy)carbonyl)amino)-3-(1-(adamantan-1-yl)-1H-1,2,3-triazol-4-yl)propanoate.*

Amorphous/oil. ^{1}H NMR ($CDCl_3$); δ 7.75 (2H, m, 4′,5′H), 7.60 (2H, m, 1′,8′H), 7.43-7.35 (3H, m, 3′,6′H, NCH), 7.35-7.28 (2H, m, 2′,7′H), 6.01 (1H, d, ^3J = 8.3, NH), 4.70 (1H, m, 2H), 4.36 (2H, m, 9′-CH_2), 4.22 (1H, m, 9′H), 3.74 (3H, s, OCH_3), 3.29 (3H, m, 3-CH_2), 2.27-2.10 (6H, m, 2″,8″,9″H), 1.85-1.60 (9H, m, 3″-7″,10″H). ^{13}C NMR ($CDCl_3$); δ 171.7, 156.0, 143.9, 143.8, 141.6, 141.2, 127.7, 127.1, 125.2, 119.9, 118.4, 67.2, 59.7, 53.5, 52.6, 47.1, 42.9, 41.5, 35.9, 35.8, 29.8, 29.5. IR (neat): ν = 3315, 2908, 2362, 2086, 1751, 1516, 1488, 1214, 1085. LRMS (APCI): 527 (100, $[M+H]^+$). HRMS (ESI): $C_{31}H_{35}N_4O_4$ calcd. $[M+H]^+$ = 527.2653, found: 527.2641.

20 *methyl (S)-2-((((9H-fluoren-9-yl)methoxy)carbonyl)amino)-3-(1-(4-cyanobenzyl)-1H-1,2,3-triazol-4-yl)propanoate.*

M.P. 162 °C. ^{1}H NMR ($CDCl_3$); δ 7.75 (2H, m, 4′,5′H), 7.63-7.53 (2H, m, 1′,8′,3″,5″H), 7.44-7.36 (2H, m, 3′,6′H), 7.32-7.23 (5H, m, 2′,7′,2″,6″H,NCH), 5.78 (1H, d, ^3J = 8.2, NH), 5.56 (1H, d, ^2J = 15.6, Ph-C$\mathbf{H_A}$$H_B$), 5.50 (1H, d, ^2J = 15.6, Ph-C$H_A$$\mathbf{H_B}$), 4.69 (1H, m, 2H), 4.36 (2H, m, 9′-CH_2), 4.19 (1H, m, 9′H), 3.73 (3H, s, OCH_3), 3.28 (2H, m, 3-CH_2). ^{13}C NMR ($CDCl_3$); δ 171.5, 155.8, 143.8, 143.7, 141.3, 139.8, 132.8, 128.1, 127.7, 127.1, 125.1, 122.3, 120.01, 120.00, 118.0, 112.7, 67.1, 53.4, 53.3, 52.7, 47.1, 28.3. IR (neat): ν = 3420, 2953, 2362, 1705, 1517, 1343, 1210, 1049. LRMS (APCI): 508 (100, $[M+H]^+$). HRMS (ESI): $C_{29}H_{26}N_5O_4$ calcd. $[M+H]^+$ = 508.1979, found: 508.1967.

21 *methyl (S)-2-((((9H-fluoren-9-yl)methoxy)carbonyl)amino)-3-(1-(4-chlorobenzyl)-1H-1,2,3-triazol-4-yl)propanoate.*

M.P. 129 °C. ^{1}H NMR (CDCl$_3$); δ 7.75 (2H, m, 4′,5′H), 7.57 (2H, m, 1′,8′H), 7.39 (2H, m, 3′,6′H), 7.32-7.25 (5H, m, 2′,7′,3″,5″H,NCH), 7.14 (2H, m, 2″,6″H), 5.84 (1H, d, ^3J = 7.3, NH), 5.47 (1H, d, ^2J = 15.2, Ph-C$\mathbf{H_A}$H$_B$), 5.41 (1H, d, ^2J = 15.2, Ph-CH$_A\mathbf{H_B}$), 4.68 (1H, m, 2H), 4.35 (2H, m, 9′-CH$_2$), 4.20 (1H, m, 9′H), 3.72 (3H, s, OCH$_3$), 3.27 (2H, m, 3-CH$_2$). ^{13}C NMR (CDCl$_3$); δ 171.8, 155.9, 143.8, 143.7, 141.3, 134.9, 132.8, 129.3, 129.2, 127.7, 127.1, 125.1, 122.1, 120.0, 67.1, 53.5, 53.4, 52.7, 47.1, 28.2. IR (neat): v = 3351, 2362, 1747, 1694, 1520, 1217, 1028. LRMS (APCI): 517 (100, [M+H]$^+$), 519 (37, [M+2+H]$^+$). HRMS (ESI): $C_{28}H_{26}ClN_4O_4$ calcd. [M+H]$^+$ = 517.1637, found: 517.1625.

22 *1-benzyl-4-(cyclohex-1-en-1-yl)-1H-1,2,3-triazole.*

Amorphous solid. ^{1}H NMR (CDCl$_3$); δ 7.35-7.19 (5H, m, Ph), 7.23 (1H, s, 5-H), 6.45 (1H, m, 2′H), 5.46 (2H, s, 1-CH$_2$), 2.31 (2H, m, 3′H), 2.15 (2H, m, 6′H), 1.70 (2H, m, 5′H), 1.62 (2H, m, 4′H). ^{13}C NMR (CDCl$_3$); δ 149.9, 134.9, 129.0, 128.6, 127.9, 127.2, 125.1, 118.2, 54.0, 26.3, 25.2, 22.4, 22.2. IR (neat): v = 2955, 2917, 2850. 2362, 1211, 1052. LRMS (APCI): 240 (100, [M+H]$^+$). HRMS (ESI): $C_{15}H_{18}N_3$ calcd. [M+H]$^+$ = 240.1495, found: 240.1497.

23 *methyl (S)-2-((((9H-fluoren-9-yl)methoxy)carbonyl)amino)-3-(1-(2-fluorobenzyl)-1H-1,2,3-triazol-4-yl)propanoate.*

M.P. 148 °C. ^{1}H NMR (CDCl$_3$); δ 7.75 (2H, m, 4′,5′H), 7.58 (2H, m, 1′,8′H), 7.43-7.26 (6H, m, 2′,3′,6′, 7′H,4″H,NCH), 7.20 (2H, m, 3″H), 7.05-7.15 (2H, m, 5″,6″H), 5.86 (1H, d, ^3J = 8.0, NH), 5.54 (2H, s, N-CH$_2$), 4.69 (1H, m, 2H), 4.35 (2H, d, ^3J = 7.3, 9′-CH$_2$), 4.20 (1H, t, ^3J = 7.3, 9′H), 3.70 (3H, s, OCH$_3$), 3.26 (2H, m, 3-CH$_2$). ^{13}C NMR (CDCl$_3$); δ 171.5, 161.7, 155.9, 143.8, 141.3, 130.9, 130.4, 127.7, 127.1, 125.2, 124.8, 121.9, 121.8, 120.0, 115.9, 115.7, 67.1, 53.4, 52.6, 47.7, 47.1, 28.2. IR (neat): v = 3368, 3070, 2632, 1749, 1686, 1515, 1217, 1040. LRMS (APCI): 501 (100, [M+H]$^+$). HRMS (ESI): $C_{28}H_{26}FN_4O_4$ calcd. [M+H]$^+$ = 501.1920, found: 501.1933.

24 *methyl (S)-2-((((9H-fluoren-9-yl)methoxy)carbonyl)amino)-3-(1-(2-methylbenzyl)-1H-1,2,3-triazol-4-yl)propanoate.*

M.P. 144°C. ^{1}H NMR (CDCl$_3$); δ 7.75 (2H, m, 4′,5′H), 7.58 (2H, m, 1′,8′H), 7.39 (6H, m, 3′,6′H), 7.34-7.26 (3H, m, 2′,7′H,NCH), 7.24-7.15 (2H, m, 3″,5″H), 7.05-7.15 (2H, m, 4″,6″H), 5.87 (1H, d, ^3J = 8.8, NH), 5.52 (1H, d, ^2J = 15.6, Ph-C$\mathbf{H_A}$H$_B$), 5.47 (1H, d, ^2J = 15.6, Ph-CH$_A\mathbf{H_B}$), 4.68 (1H, m, 2H), 4.34 (2H, m, 9′-CH$_2$), 4.20 (1H, m, 9′H), 3.70 (3H, s, OCH$_3$), 3.24 (2H, m, 3-CH$_2$), 2.24 (3H, s, 2″CH$_3$). ^{13}C NMR (CDCl$_3$); δ 171.6, 155.9, 143.9, 143.8, 141.3, 136.9, 132.6, 131.0, 129.2, 129.1, 127.7, 127.1, 126.6, 125.2, 119.9, 67.2, 52.5, 52.4, 47.1, 28.2, 18.9. IR (neat): v = 2929, 2355, 1717, 1506, 1197, 1058. LRMS (APCI): 497 (100, [M+H]$^+$). HRMS (ESI): $C_{29}H_{29}N_4O_4$ calcd. [M+H]$^+$ = 497.2183, found: 497.2169.

25 *1-benzyl-4-(2,4-dichlorophenyl)-1H-1,2,3-triazole.*

M.P. 137 °C. ^{1}H NMR (CDCl$_3$); δ 8.21 (1H, d, ^3J = 8.5, 6'-H), 8.09 (1H, s, 5-H), 7.45 (1H, d, ^4J = 2.0, 3'-H), 7.40-7.27 (6H, m, 5'-,2"-6"-H), 5.61 (2H, s, 1-CH$_2$). ^{13}C NMR (CDCl$_3$); δ 143.3, 134.8, 134.1, 130.5, 129.9, 129.2, 128.8, 128.0, 127.6, 123.1, 54.3. IR (neat): v = 3731, 2362, 1652, 1456, 1055. LRMS (APCI): 304 (100, [M+H]$^+$), 306 (65, [M+2+H]$^+$), 308 (10, [M+4+H]$^+$). HRMS (ESI): $C_{15}H_{12}Cl_2N_3$ calcd. [M+H]$^+$ = 304.0403, found: 304.0406.

Table S2: Binding inhibition IC_{50} of FITC-NoxaB to Mcl-1 of small molecules not tested against cell lines.

	Structure	FA IC_{50} (nM)		Structure	FA IC_{50} (nM)
**S8**	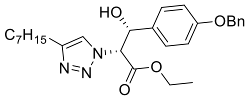	237 ± 40	**S14**	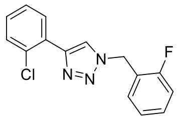	1370 ± 938
**S9**	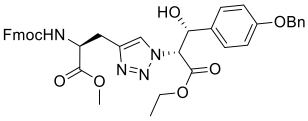	266 ± 125	**S15**	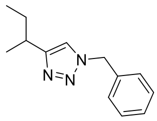	3310 ± 488
**S10**	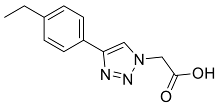	497 ± 367	**S16**	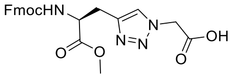	5280 ± 1690
**S11**	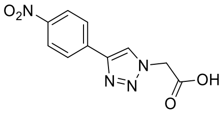	561 ± 130	**S17**	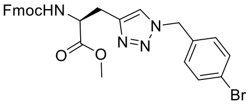	>100000
**S12**	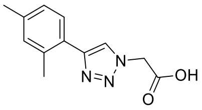	766 ± 484	**S18**	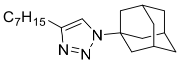	>100000
**S13**	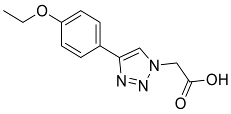	1320 ± 1100	**S19**	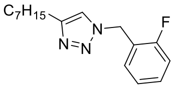	>100000

Fmoc, 9-fluorenylmethylcarbony. IC_{50} determined by non-linear regression of at least three experiments. Errors are the transformed greater extreme of the standard error.

4. Fluorescence Anisotropy Binding Assay

Fluorescence anisotropy was carried out on a BMG Labtech clariostar microplate reader with a fluorescence anisotropy optic measuring at 482/530 nm. Low-binding, Corning 96-well black plates were used and all reagents used in the assay were biological grade and purchased from Sigma Aldrich and Novabiochem. All solutions were made using MilliQ water.

Anisotropy values were automatically calculated using the following formula

$$r = \frac{l_a - l_b}{l_a + 2l_b}$$

$$I = l_a + 2l_b$$

Where r = anisotropy, l_a = parallel emission light, l_b = perpendicular emission light and I = total intensity.

K_D was determined using GraphPad Prism Version 6.0 software, using the One Site – Total binding linear regression model

$$y = \frac{B_{max} \times x}{K_D + x} + NS \times x$$

Where B_{max} is the maximum specific binding, K_D is the equilibrium binding constant, and NS is the slope of nonspecific binding.

For ideal conditions, the concentration of receptor should be fixed at between K_D and 80% B_{max}.

IC_{50} was determined using GraphPad Prism Version 6.0 software, using a four-parameter logistic model, according to precedent from Ottmann and co-workers.[5] Errors are the transformed greater extreme of the standard error.

$$y = r_{min} + \frac{(r_{max} - r_{min})}{1 + 10^{((logIC_{50} - x) \times Hill\ Slope)}}$$

Mcl-1

For binding each well contained 10 µL of Mcl-1 protein in 10-fold dilutions (0.37 pM to 3.7 µM) and 90 µL of 5 nM fluorescently-tagged high affinity binding peptide FITC-NoxaB. Peptide and protein were dissolved in PBS: 0.05% Tween-20 buffer at pH 7.4. Reagents were incubated for 30 min at room temperature prior to reading. Bubbles were removed and 8 data points were generated. Nonlinear regression was then used to process the data, giving the K_D of the fluorescently-tagged peptide to be 5.2 nM. Performed in triplicate.

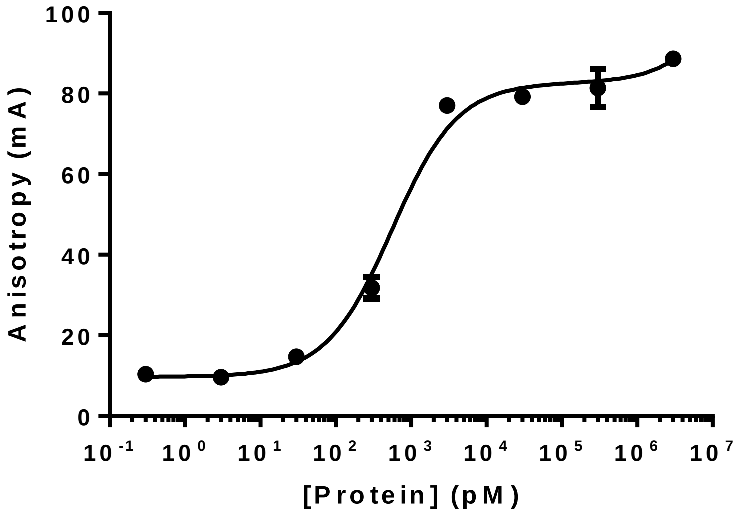

Figure S 1: Fluorescence Anisotropy direct titration. Fraction bound of FITC-NoxaB on Mcl-1

A titration of 100 nM Mcl-1 with FITC-NoxaB suggested that with a 100 µL well volume 5 nM FITC-NoxaB peptide produced the greatest change in polarisation. To confirm reproducibility of data, a Z-prime test was undertaken, in which 24 wells containing 100 µM NoxaB (Ac-AAQLRRIGDKVNLRQKLLN-NH_2) and 24 wells containing DMSO were screened. The means (µ) and standard deviations (σ) were inputted into the following equation:

$$Z' = 1 - \frac{3(\sigma p + \sigma n)}{|\mu p - \mu n|}$$

The Z-prime produced a value of 0.78, which means that the assay gives excellent reproducibility.

Inhibition screens were carried out using 10 nM Mcl-1, 5 nM fluorescently-tagged peptide and 100 µM of inhibitor. Compounds were dissolved in DMSO. A positive control (NoxaB) and negative control (DMSO) were used to define the minimum and maximum values and ensure the assay was performing adequately. Compounds which demonstrated inhibition by fluorescence anisotropy but also demonstrated excessive fluorescence activity were excluded. Compounds showing inhibition

were then diluted in 10-fold dilutions (from 100 µM to 10 pM well concentration) to generate IC_{50} values, in duplicate. The IC_{50} of NoxaB was calculated to be 0.65 µM.

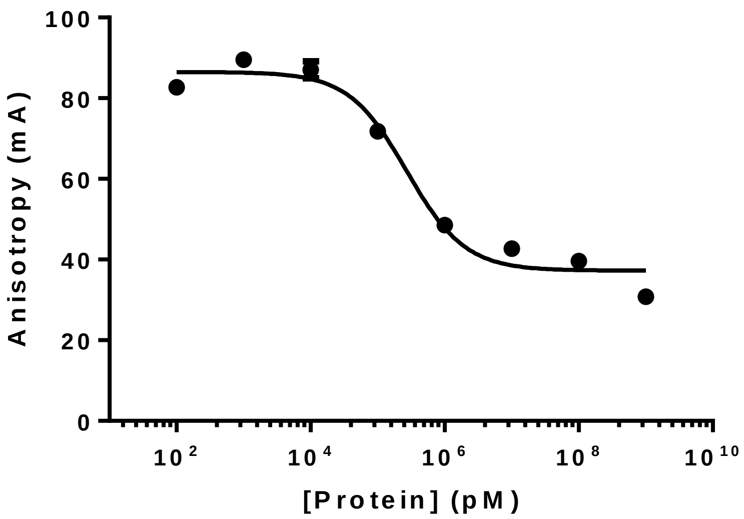

Figure S 2: Fluorescence Anisotropy dose response titration of NoxaB vs FITC-NoxaB on Mcl-1

Bcl-2

For binding each well contained 10 µL of Bcl-2 protein in 10-fold dilutions (0.90 pM to 9.0 µM) and 90 µL of 5 nM fluorescently-tagged high affinity binding peptide FITC-Bid. Peptide and protein were dissolved in PBS: 0.05% Tween-20 buffer at pH 7.4. Reagents were incubated for 30 min at room temperature prior to reading. Bubbles were removed and 8 data points were generated. Nonlinear regression was then used to process the data, giving the K_D of the fluorescently-tagged peptide to be 15.4 nM. $B_{max} \approx$ 90-100 nM. Performed in triplicate.

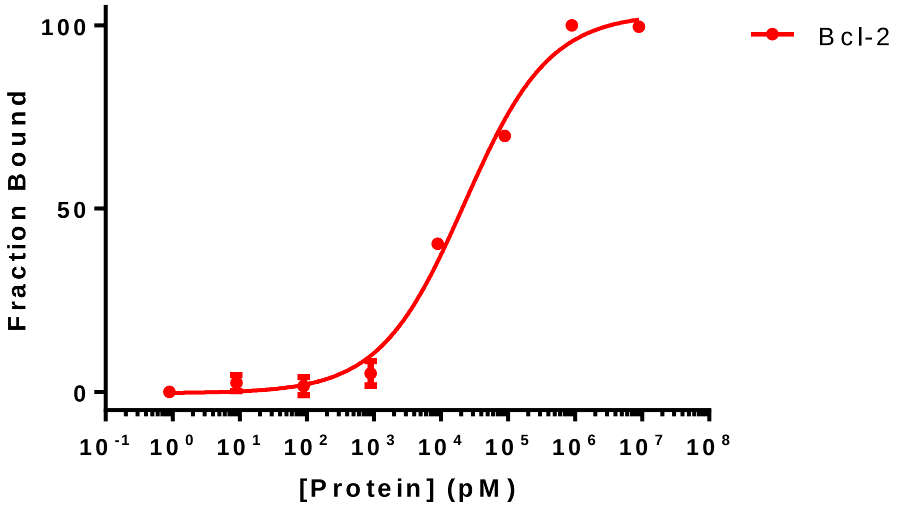

Figure S 3: Fluorescence Anisotropy direct titration. Fraction bound of FITC-Bid on Bcl-2. Normalized

A titration of 100 nM Bcl-2 with FITC-Bid suggested that with a 100 µL well volume 5 nM FITC-Bid peptide produced the greatest change in polarisation. Inhibition screens were carried out using

30 nM Bcl-2, 5 nM fluorescently-tagged peptide and 100 µM of inhibitor. Compounds were dissolved in DMSO. A positive control (ABT-263, purchased from Selleckchem) and negative control (DMSO) were used to define the minimum and maximum values and ensure the assay was performing adequately. Compounds showing inhibition were then diluted in 10-fold dilutions (from 100 µM to 10 pM well concentration) to generate IC_{50} values, in duplicate. The IC_{50} of ABT-263 was calculated to be 349 nM.

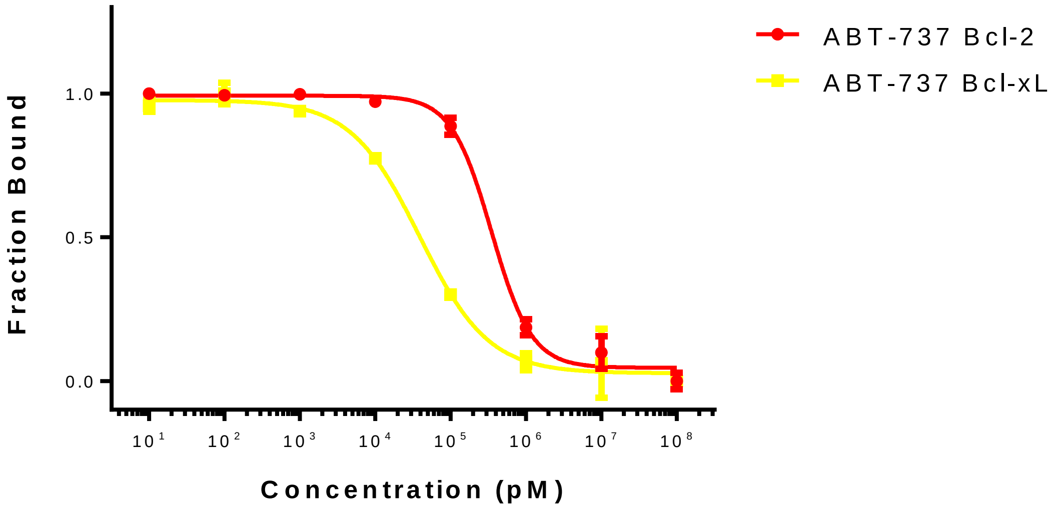

Figure S 4: Fluorescence Anisotropy dose response titration of ABT-737 vs FITC-Bid on Bcl-2 (Red) and Bcl-x_L (Yellow). Normalized

Bcl-x_L

For binding each well contained 10 µL of Bcl-x_L protein in 10-fold dilutions (0.45 pM to 4.5 µM) and 90 µL of 5 nM fluorescently-tagged high affinity binding peptide FITC-Bid. Peptide and protein were dissolved in PBS: 0.05% Tween-20 buffer at pH 7.4. Reagents were incubated for 30 min at room temperature prior to reading. Bubbles were removed and 8 data points were generated. Nonlinear regression was then used to process the data, giving the K_D of the fluorescently-tagged peptide to be 24.9 nM. B_{max} ≈ 90-100 nM. Performed in triplicate.

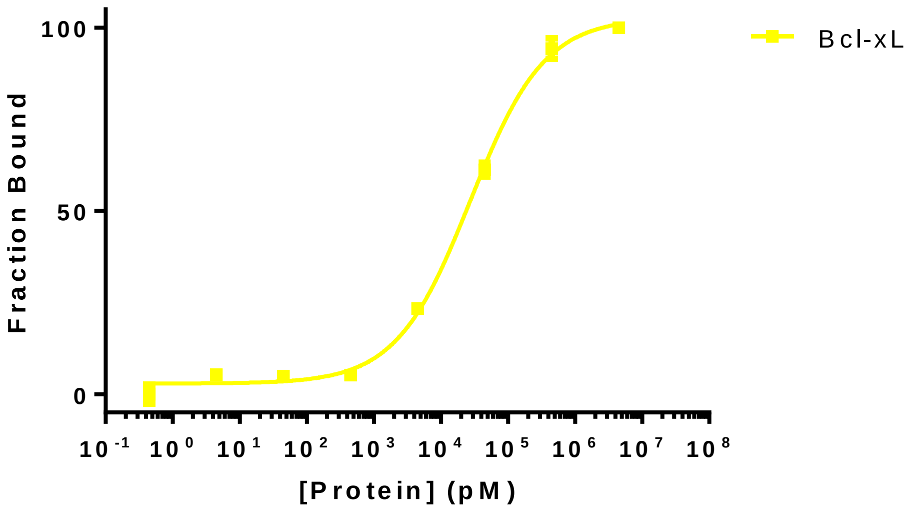

Figure S 5: Fluorescence Anisotropy direct titration. Fraction bound of FITC-Bid on Bcl-x_L. Normalized

A titration of 100 nM Bcl-x_L with FITC-Bid suggested that with a 100 µL well volume 5 nM FITC-Bid peptide produced the greatest change in polarisation. Inhibition screens were carried out using 45 nM Bcl-x_L, 5 nM fluorescently-tagged peptide and 100 µM of inhibitor. Compounds were dissolved in DMSO. A positive control (ABT-263, purchased from Selleckchem) and negative control (DMSO) were used to define the minimum and maximum values and ensure the assay was performing adequately. Compounds showing inhibition were then diluted in 10-fold dilutions (from 100 µM to 10 pM well concentration) to generate IC_{50} values, in duplicate. The IC_{50} of ABT-263 was calculated to be 39 nM.

5. Molecular Docking

The X-ray crystal structure of Mcl-1 with a modified Noxa ligand[6] (PDB ID 2NLA) was utilized for docking calculations. The ligand was removed and the protein was prepared using the Protein Preparation application from the Schrodinger Suite 2015-2.[7] The bound peptide was modified using Maestro drawing tools, and the resulting receptor was again prepared using Protein Preparation. Ligands were taken from the Sigma-Aldrich virtual library and prepared using the LigPrep application from the Schrodinger Suite 2015-2. Binding site grid generation was preformed using the Receptor Grid Generation application from the Schrodinger 2015-2 suite using the using the pocket left vacant by the removed section of the peptide, using the centre point of residues Arg224, Cys231 and His263 for the alkyne ligands, or the centre point of residues Asn223, Phe318 and Val 321 for the azide ligands. Covalent docking was performed using the CovDock tool from the Schrodinger Suite 2015-2,[8] using the following custom reaction code to generate the desired azido-alkynyl condensation.

Alkyne receptor, azide ligand:

```
LIGAND_SMARTS_PATTERN 4,CN=[N+]=[N-]
RECEPTOR_SMARTS_PATTERN 2,C#CCC=O
CUSTOM_CHEMISTRY ("<1>|<2>",("bond",1,(1,2)))
CUSTOM_CHEMISTRY ("<1>#C",("bond",2,(1,2)))
CUSTOM_CHEMISTRY ("<1>[N-]=[N+]=NC",("bond",1,(3,4)))
CUSTOM_CHEMISTRY ("<1>(=C)[N-]=[N+]N",("bond",1,(2,5)))
```

Azide receptor, alkyne ligand:

```
RECEPTOR_SMARTS_PATTERN 4,CN=[N+]=[N-]
LIGAND_SMARTS_PATTERN 2,C#C
CUSTOM_CHEMISTRY ("<1>",("charge", 0, 1))
CUSTOM_CHEMISTRY ("<1>=[N+]",("charge", 0, 2))
CUSTOM_CHEMISTRY ("<1>|<2>",("bond",1,(1,2)))
CUSTOM_CHEMISTRY ("<2>#C",("bond",2,(1,2)))
CUSTOM_CHEMISTRY ("<1>=[N]=[N]",("bond",1,(2,3)))
CUSTOM_CHEMISTRY ("<1>[N]",("bond",2,(1,2)))
CUSTOM_CHEMISTRY ("<1>([C]=[C])=[N][N]",("bond",1,(3,5)))
```

Induced fit docking was performed with protein flexibility using the Induced Fit[9] application from the Schrodinger Suite 2015-2. A total of 100 possible binding conformations were generated and grouped into clusters using a 1.0 Å root-mean-square tolerance. Conformations were then ranked based on their Glide e-model scores.[10] For induced fit docking, conformations with the lowest glide-emodel score were docked again using Induced Fit to generate the lowest energy conformation. Figures were generated using the Chimera package from the Computer Graphics Laboratory, University of California, San Francisco (supported by NIH P41 RR-01081).

6. Cell Culture

The MiaPaCa-2 cell line, BxPC-3 cell line and AsPC-1 cell line were purchased from the European Collection of Cell Cultures (ECACC, Porton Down, UK). MiaPaCa-2 cells were cultured in DMEM media with 2 mM L-glutamine, 100 U/mL penicillin, 100 µg/mL streptomycin and 10% FCS. BxPC-3 cells and AsPC-1 cells were cultured in RPMI-1640 media containing 2 mM L-glutamine, 100 U/mL penicillin, 100 µg/mL streptomycin and 10% FCS. Cells were sub-cultured twice weekly and maintained at 37 °C and 5% CO_2.

7. Cellular assay

MTS Cytotoxicity Assay

The anti-proliferative activity of the compounds studied was assessed by MTS assay (Promega) following the manufacturer's instructions. Cells were seeded at 5 x 10^3 / 100 µL in a 96-well plate and left untreated or treated with DMSO (vehicle control), or compounds **1-4** (100 µM – 10 pm, in well concentration) in triplicate for 72 h at 37 °C with 5% CO_2. Following this, MTS assay reagent was added to each well and the plates incubated for 4 h at 37 °C with 5% CO_2. The absorbance was measured at 492 nm using the BMG Labtech POLARstarOPTIMA microplate reader. IC_{50} values were calculated using GraphPad Prism Version 6.0 software, using a four-parameter logistic non-linear regression model. Errors are the transformed greater extreme of the standard error.

Annexin-V/ PI staining

An Annexin-V-FLUOS/Propidium iodide staining kit (Roche Applied Science) was used to detect phosphatidylserine externalization in pancreatic cancer cell line BxPC-3. Cells, seeded in clear 96-well plates at 5 x 10^3 cells/well were treated with compounds at the determined IC_{50} for 4 hours, and treated with staining kit according to the manufacturer's protocol. Fluorescence microscopy

was used to examine cells and pictures taken with an inverted Leica DMII fluorescence microscope at 10x magnification.

Caspase 3 activation assay

Caspase 3 activation was determined using a Caspase 3 assay kit (Colorimetric, Abcam ab39401) in the pancreatic cancer cell line BxPC-3. Cells were seeded in 12 well plates at 1 x 10^5 cells/well and treated with compounds at the determined IC_{50} for 4 hours. Cells were subsequently treated according to the manufacturer's protocol. Samples were analysed using a BMG Labtech clariostar microplate reader at 405 nm. Data was analysed using GraphPad Prism Version 6.0 software.

8. NMR Spectra

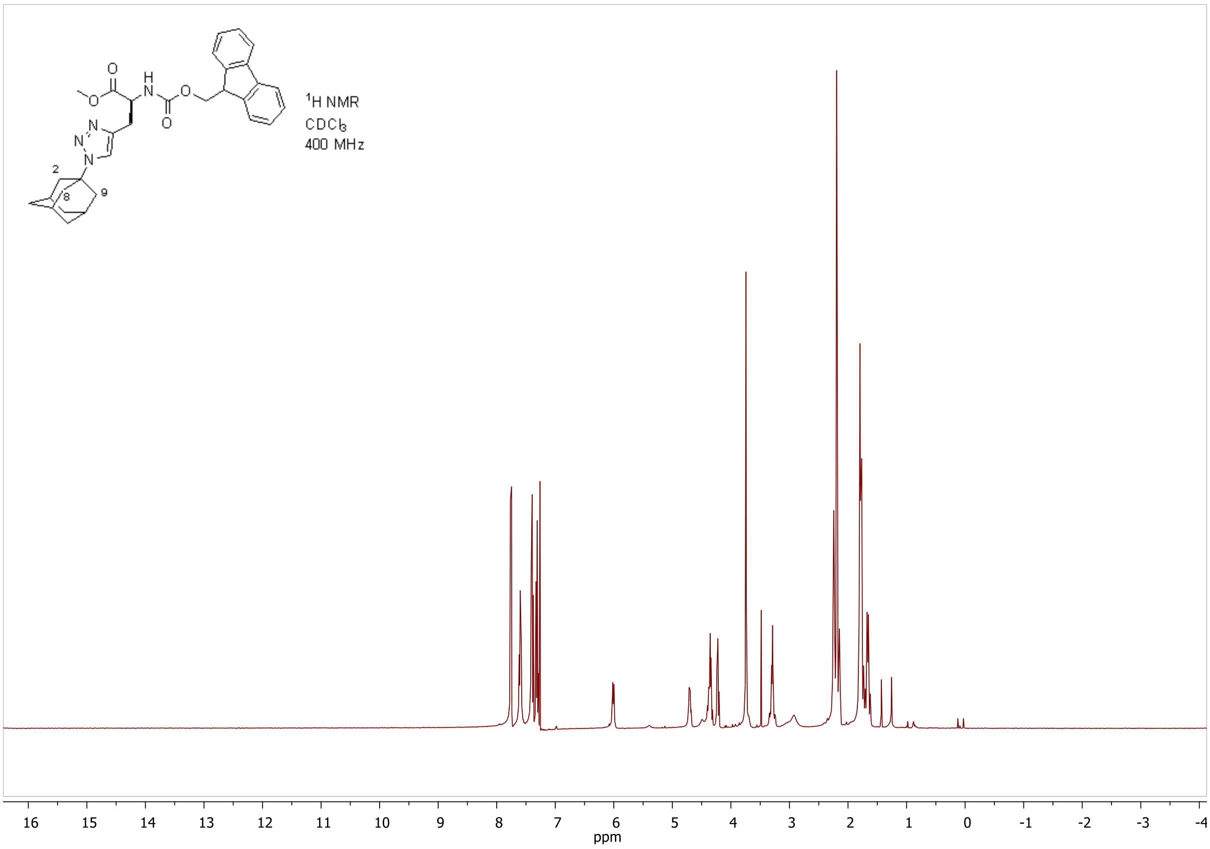

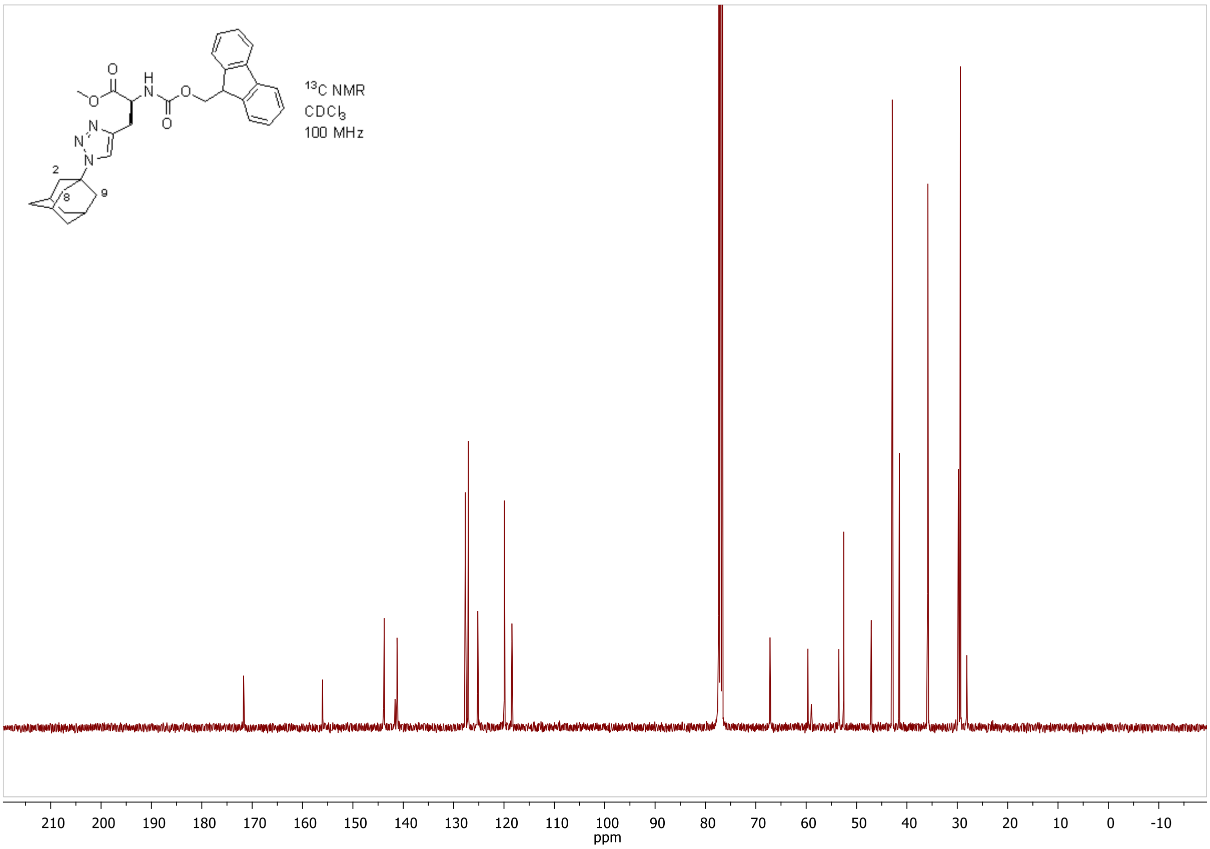

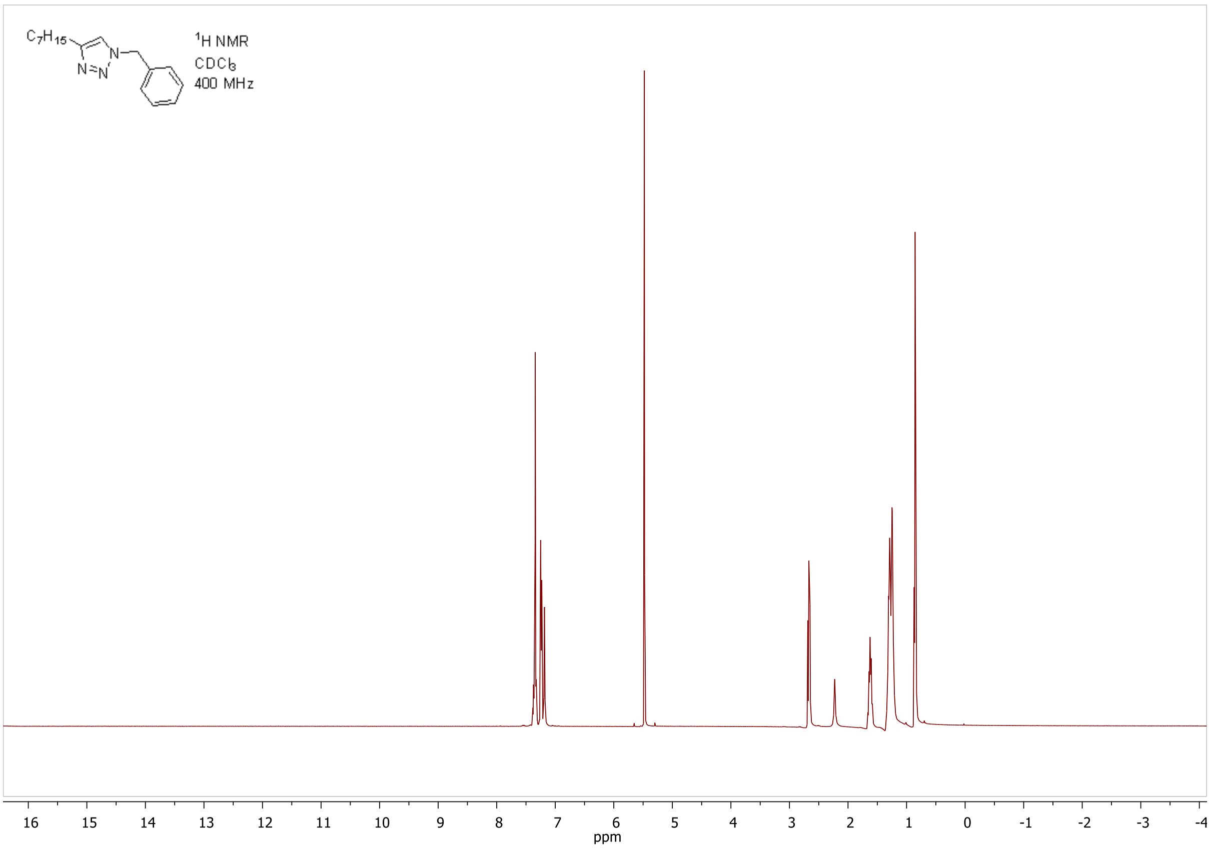

C7H15
N
N=N
1H NMR
CDCl3
400 MHz
16 15 14 13 12 11 10 9 8 7 6 5 4 3 2 1 0 -1 -2 -3 -4
ppm

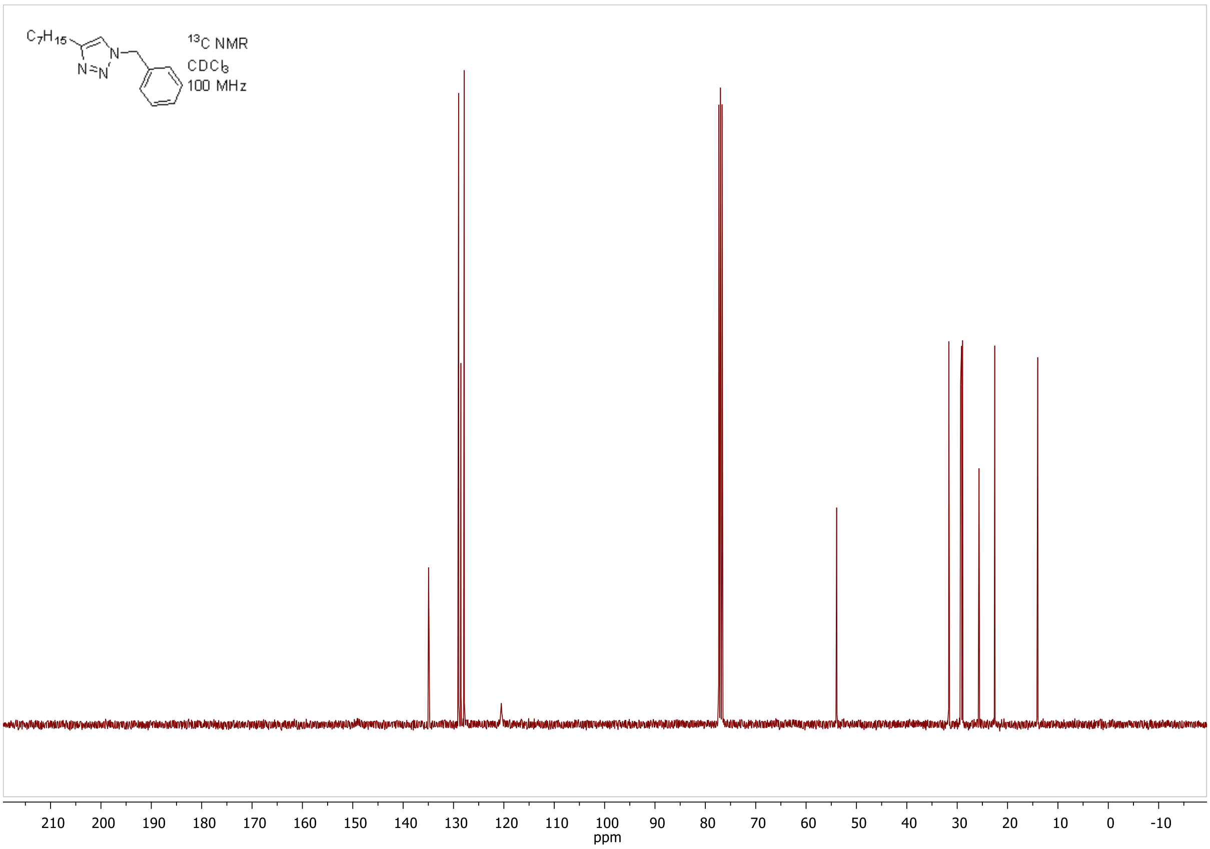

C7H15
N
N=N
13C NMR
CDCl3
100 MHz
210 200 190 180 170 160 150 140 130 120 110 100 90 80 70 60 50 40 30 20 10 0 -10
ppm

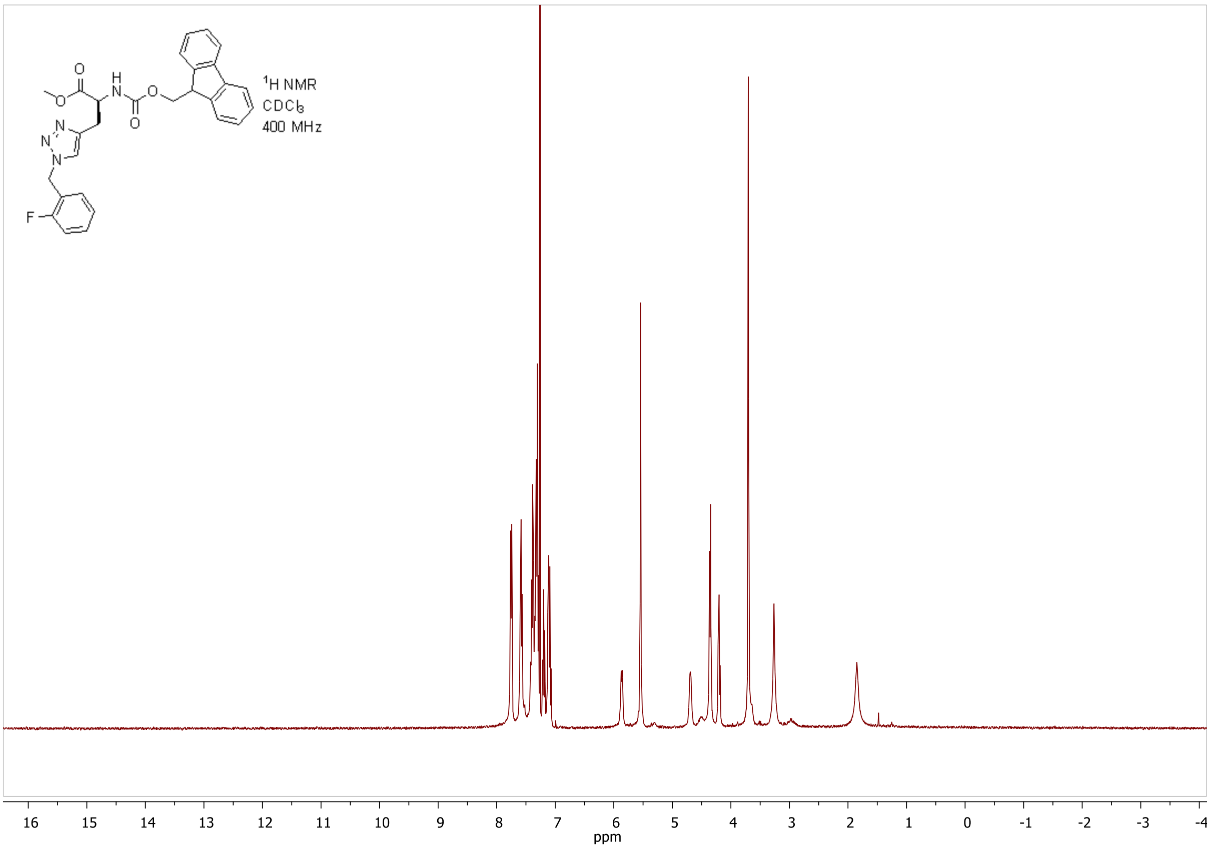

^{1}H NMR
CDCl$_3$
400 MHz
16 15 14 13 12 11 10 9 8 7 6 5 4 3 2 1 0 -1 -2 -3 -4
ppm

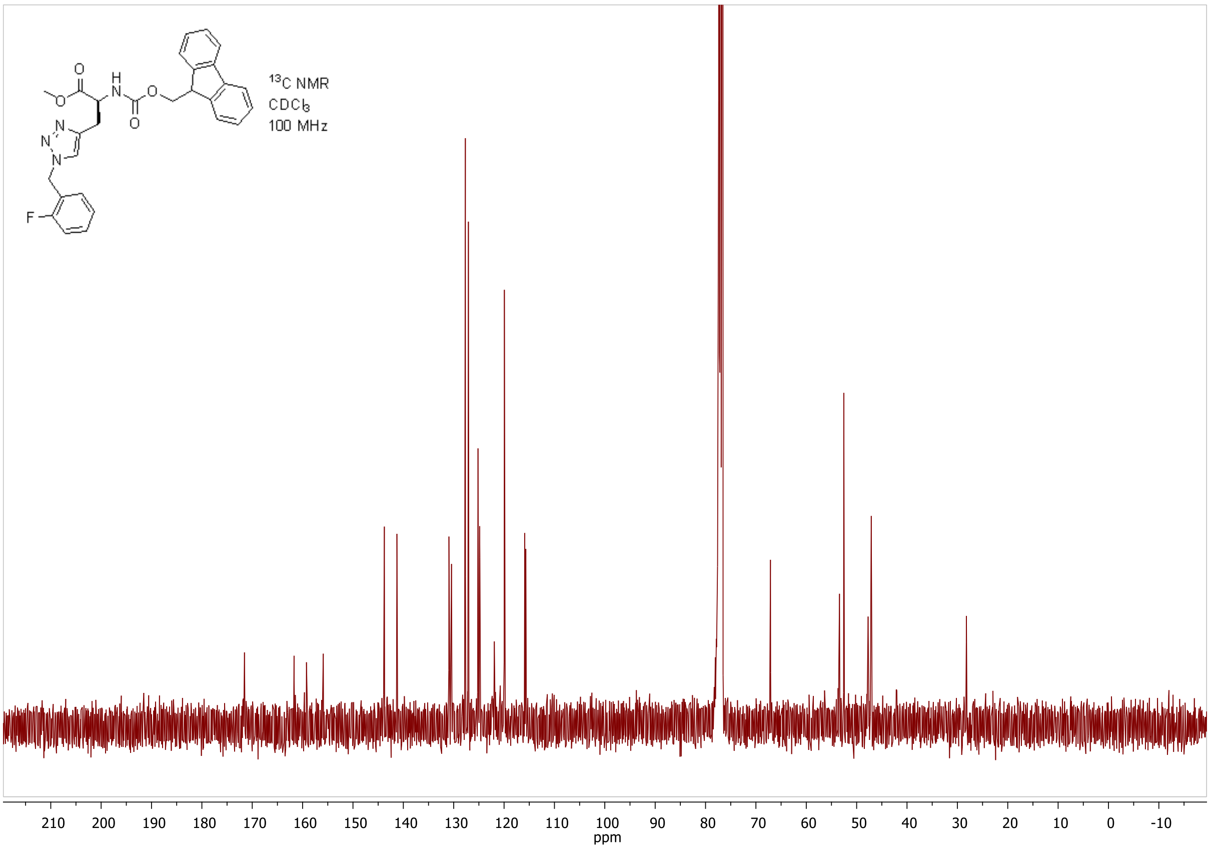

^{13}C NMR
CDCl$_3$
100 MHz
210 200 190 180 170 160 150 140 130 120 110 100 90 80 70 60 50 40 30 20 10 0 -10
ppm

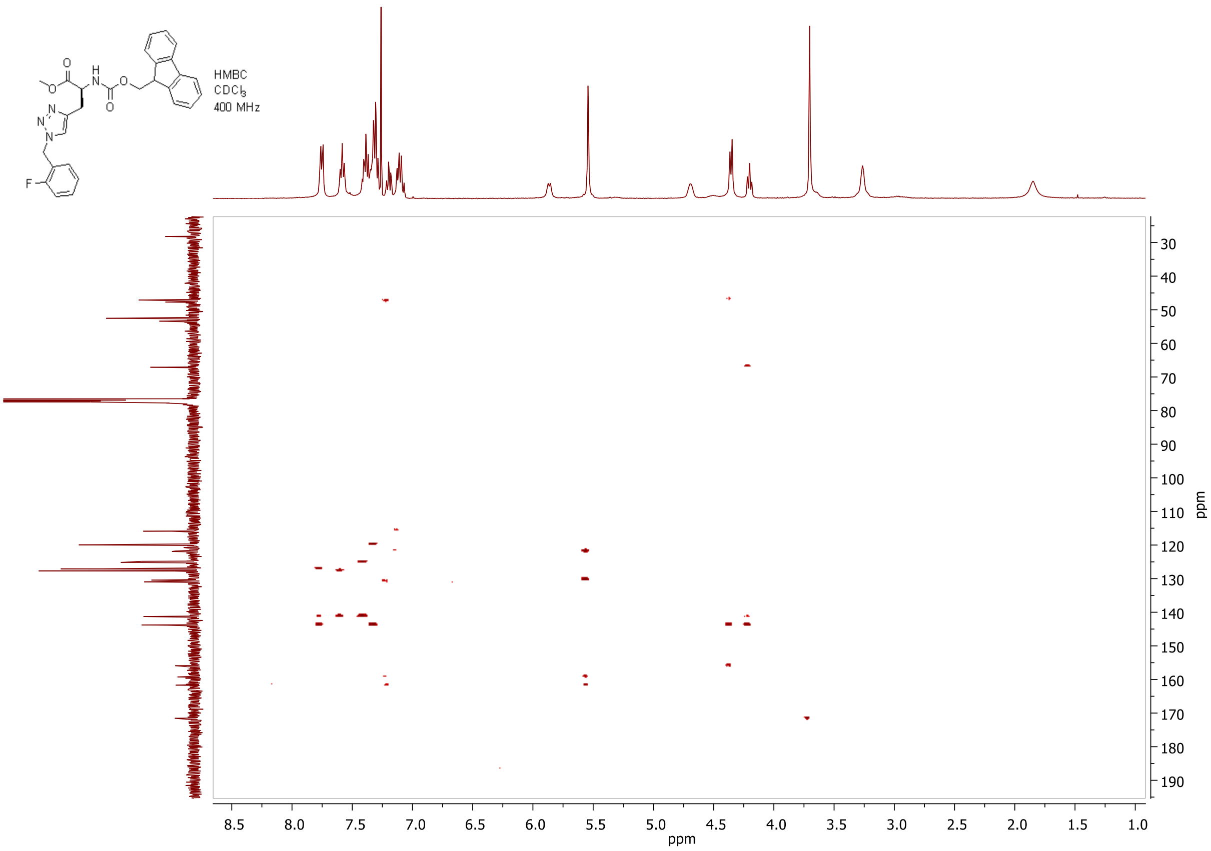
HMBC
CDCl3
400 MHz
ppm
ppm

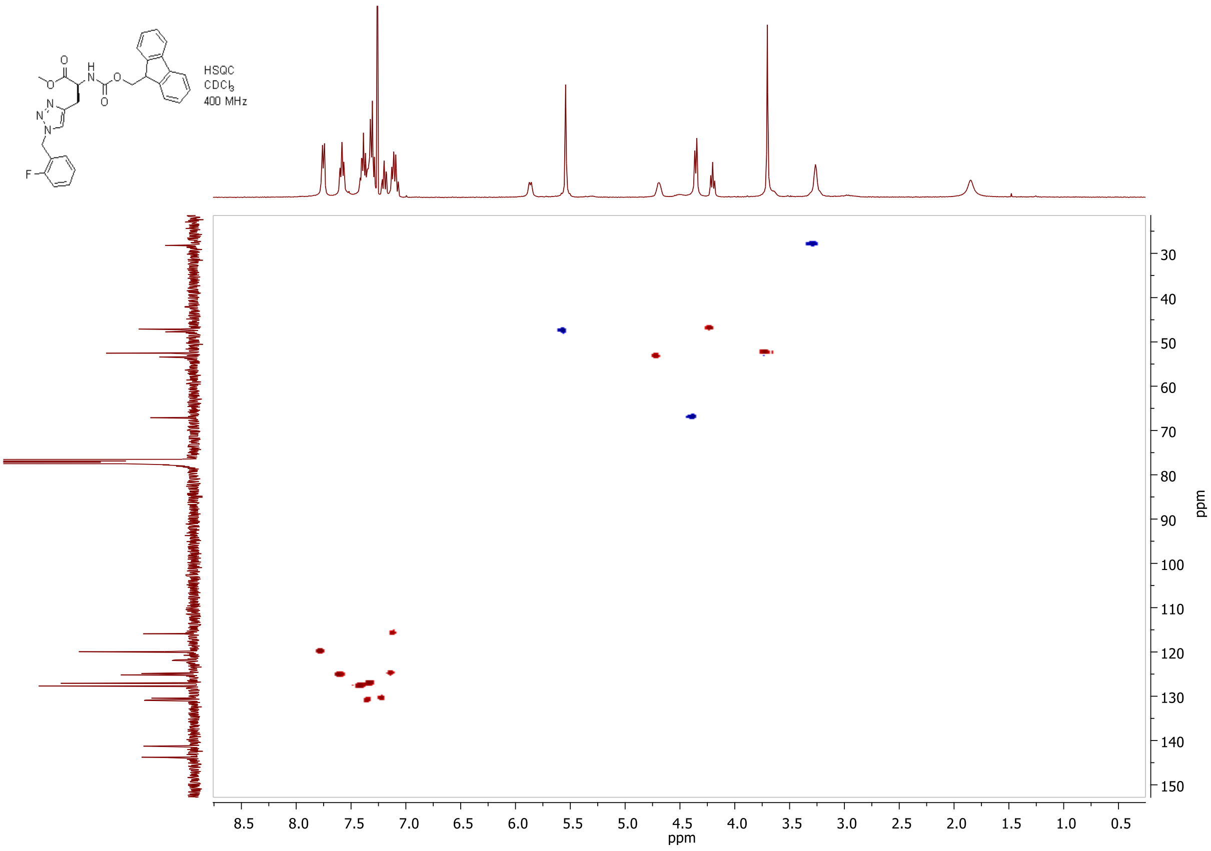
HSQC
CDCl3
400 MHz
ppm
ppm

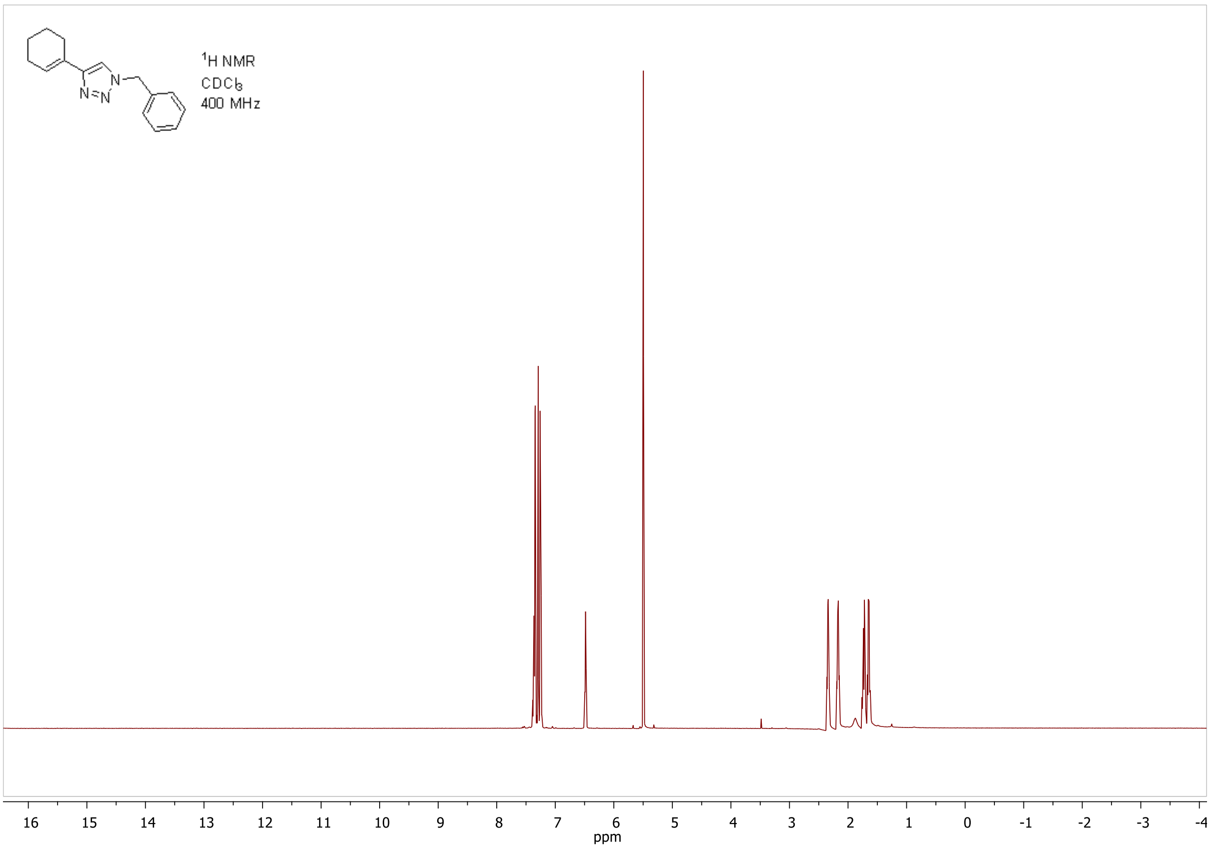

N
N
N
^{1}H NMR
$CDCl_3$
400 MHz
16 15 14 13 12 11 10 9 8 7 6 5 4 3 2 1 0 -1 -2 -3 -4
ppm

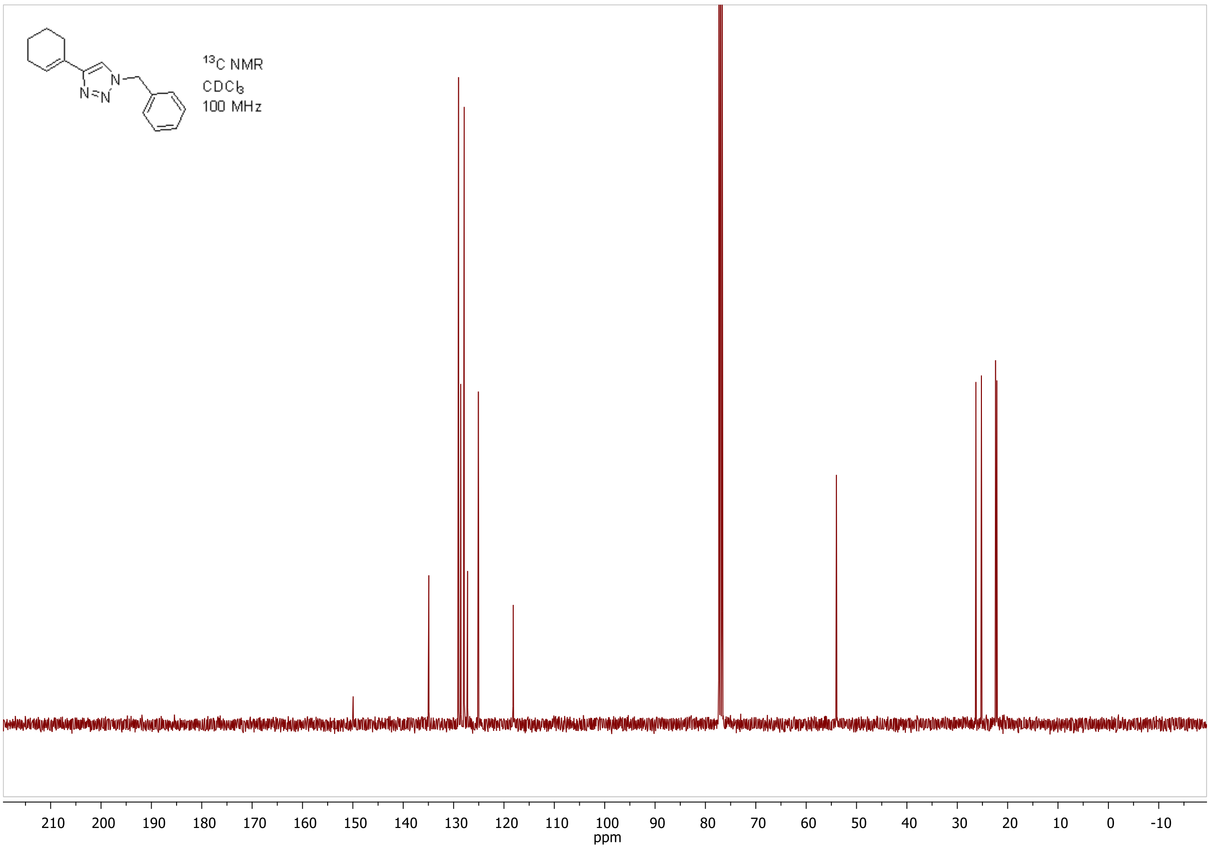

N
N
N
^{13}C NMR
$CDCl_3$
100 MHz
210 200 190 180 170 160 150 140 130 120 110 100 90 80 70 60 50 40 30 20 10 0 -10
ppm

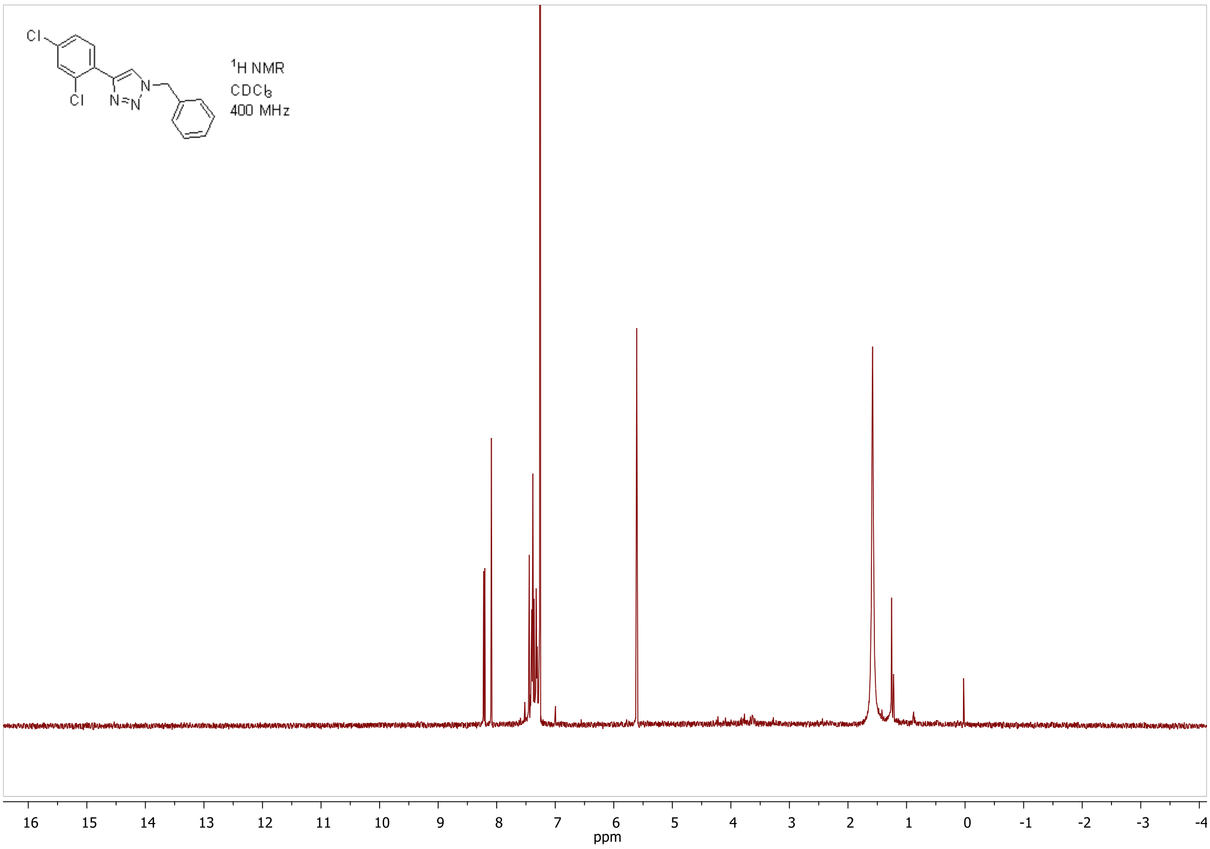

Cl
Cl
N
N=N
^{1}H NMR
CDCl3
400 MHz
16 15 14 13 12 11 10 9 8 7 6 5 4 3 2 1 0 -1 -2 -3 -4
ppm

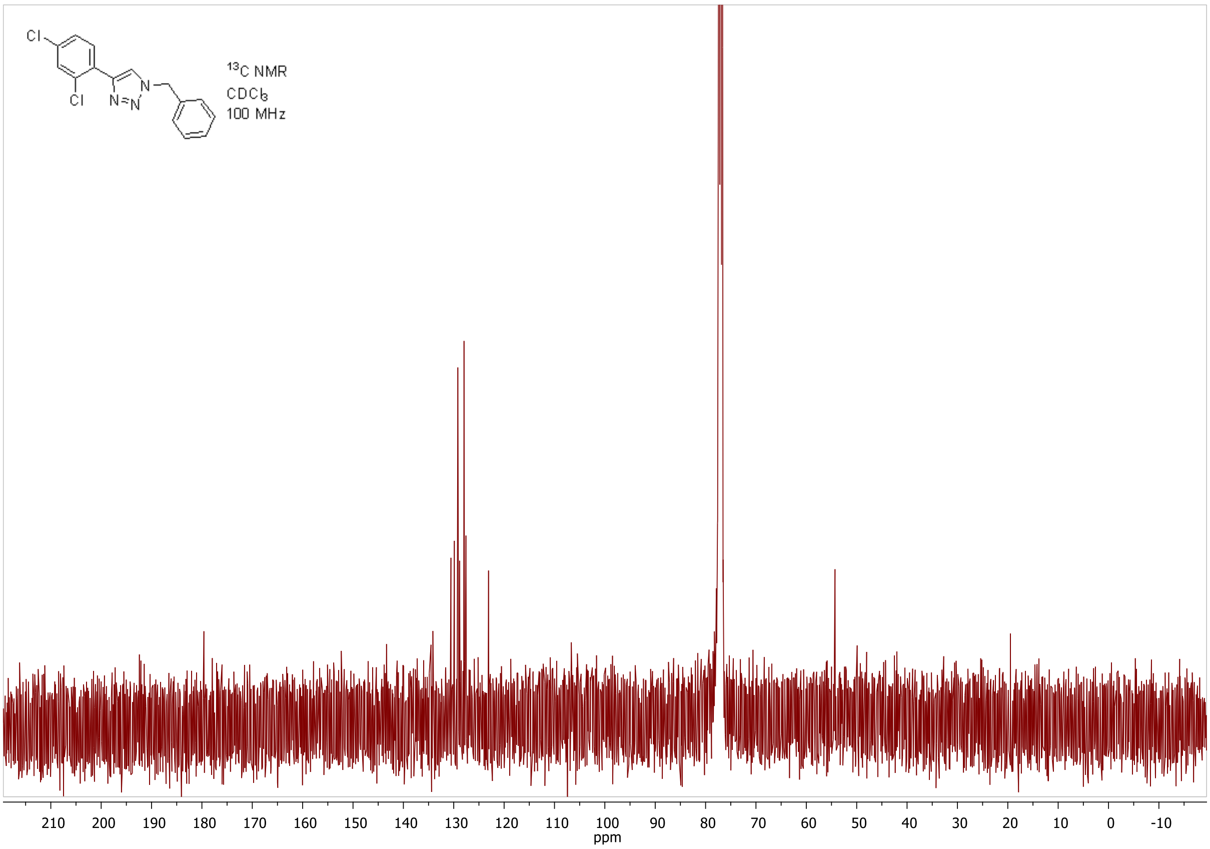

Cl
Cl
N
N=N
^{13}C NMR
CDCl3
100 MHz
210 200 190 180 170 160 150 140 130 120 110 100 90 80 70 60 50 40 30 20 10 0 -10
ppm

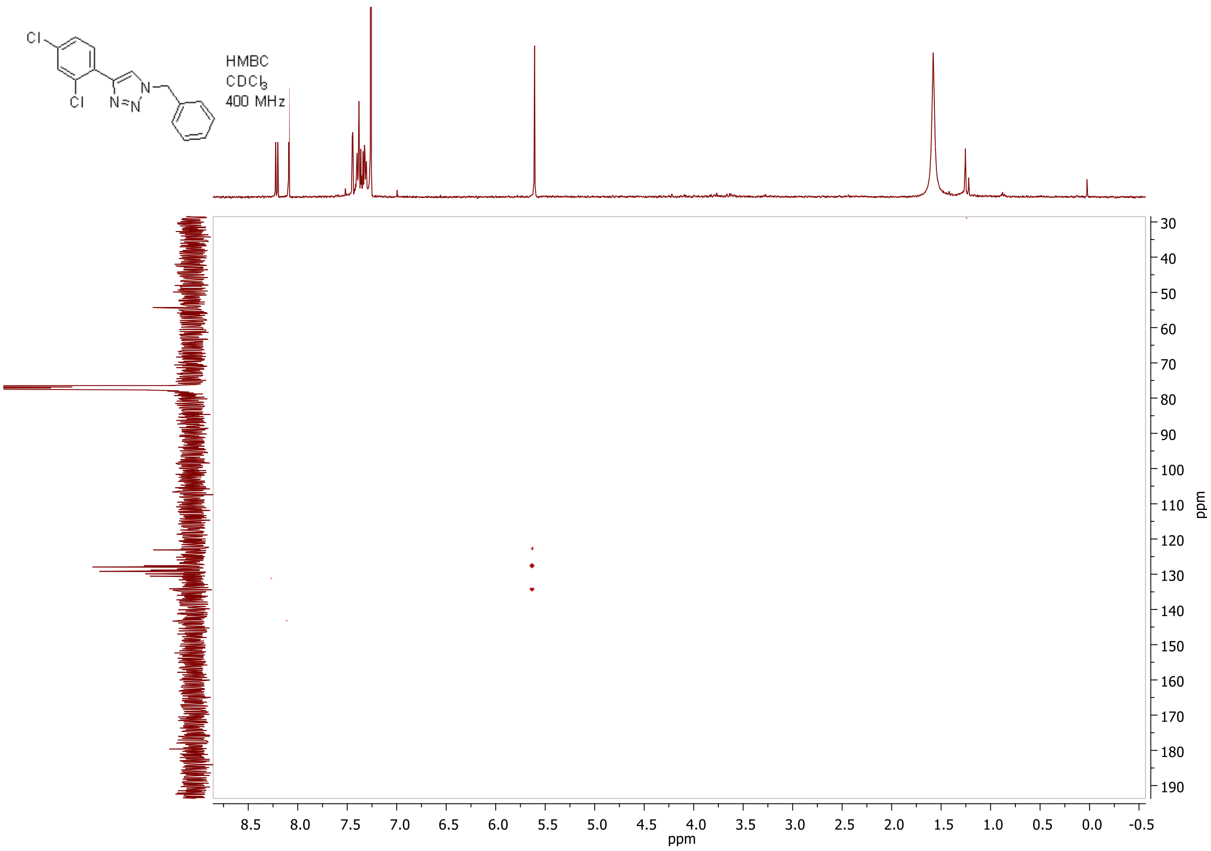

Cl
Cl
N
N=N
HMBC
CDCl3
400 MHz
ppm
ppm

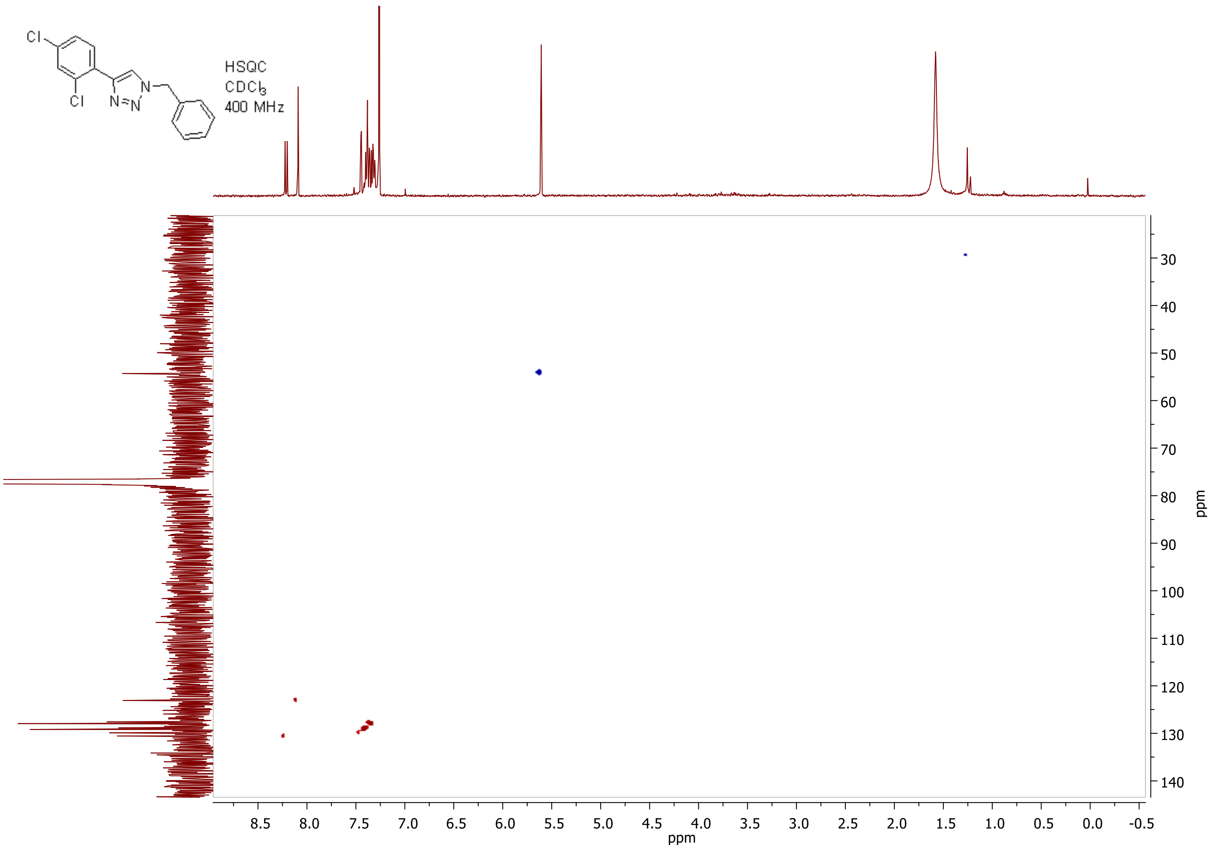

Cl
Cl
N
N=N
HSQC
CDCl3
400 MHz
ppm
ppm

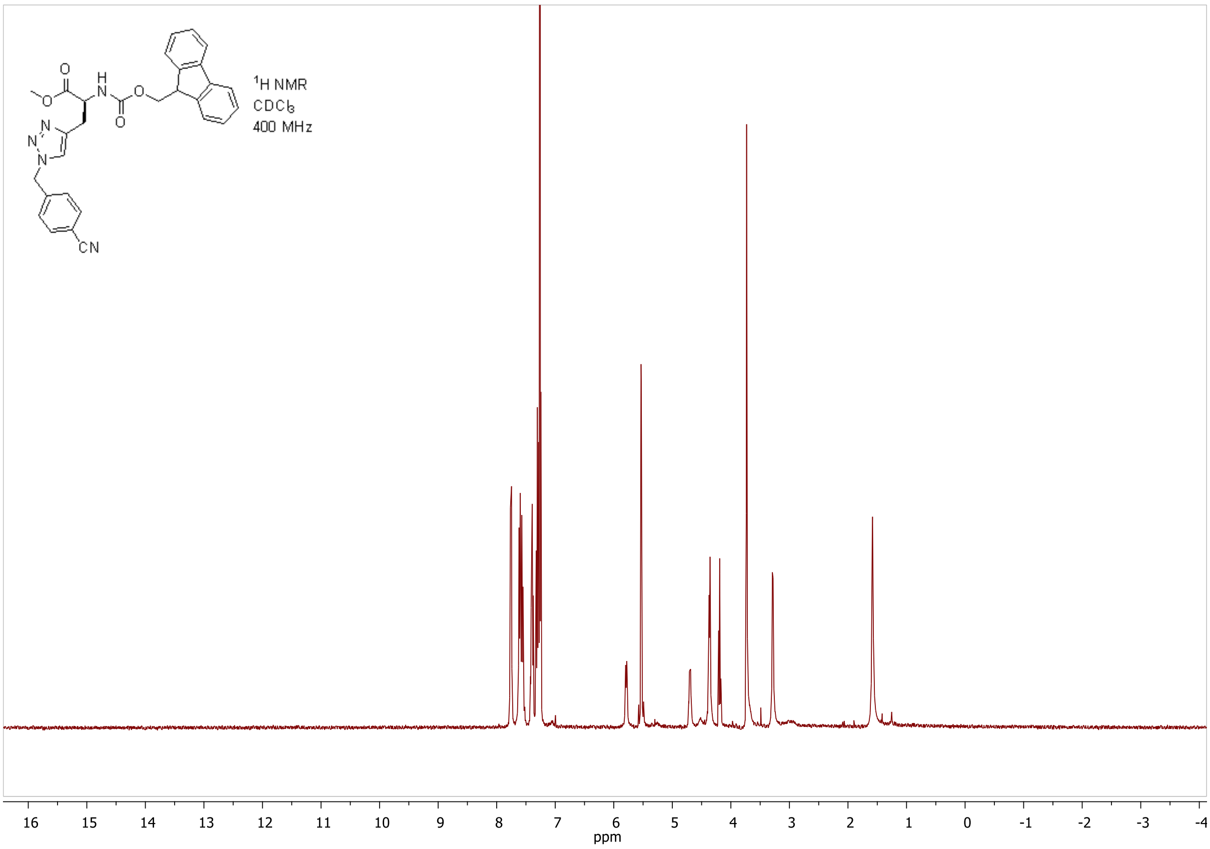

1H NMR
CDCl3
400 MHz
16 15 14 13 12 11 10 9 8 7 6 5 4 3 2 1 0 -1 -2 -3 -4
ppm

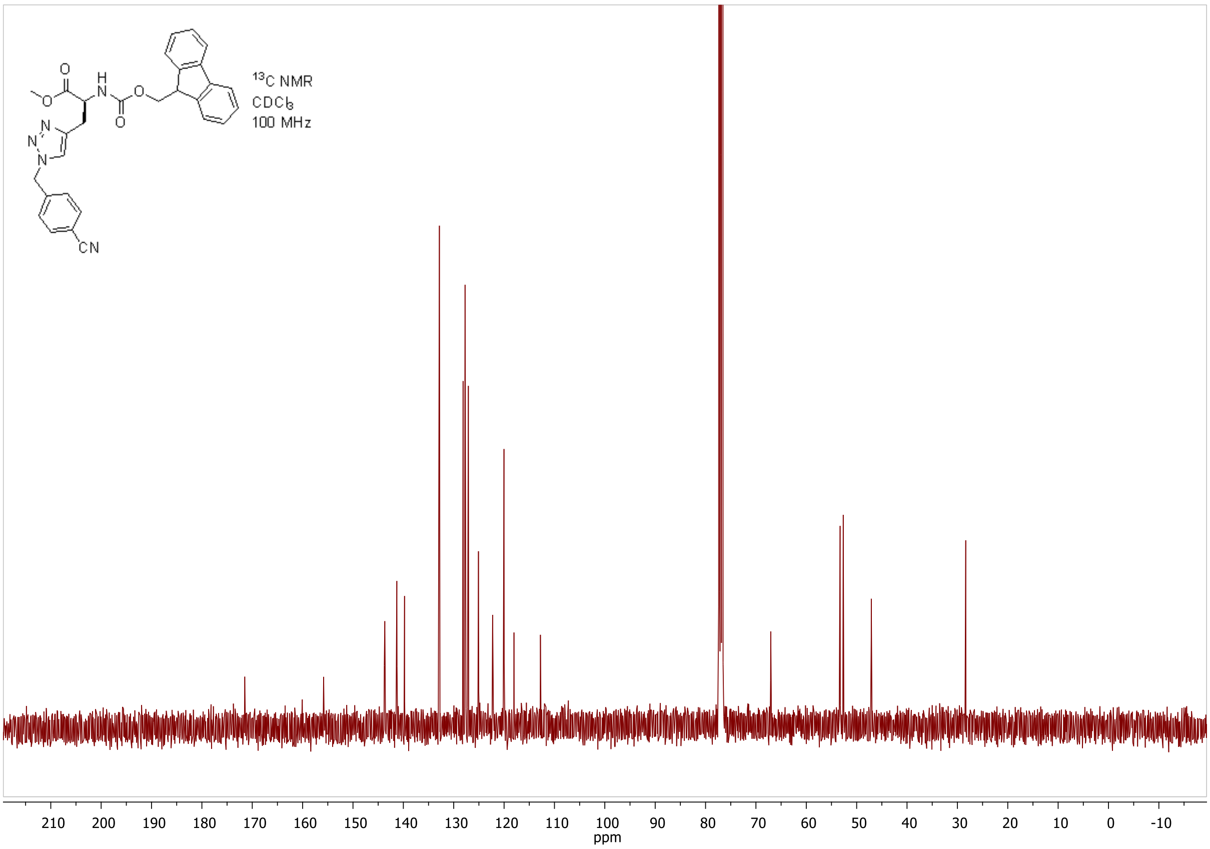

13C NMR
CDCl3
100 MHz
210 200 190 180 170 160 150 140 130 120 110 100 90 80 70 60 50 40 30 20 10 0 -10
ppm

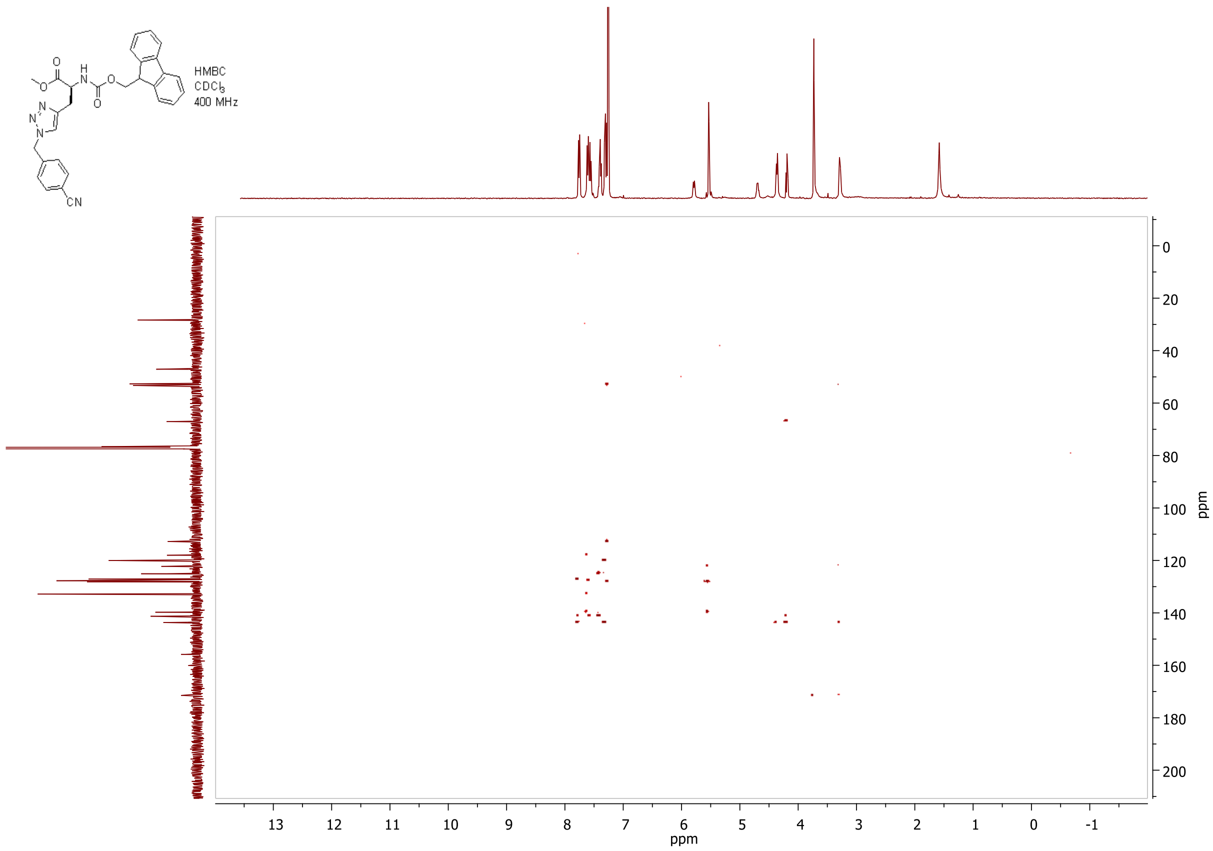
HMBC
CDCl3
400 MHz
ppm
ppm

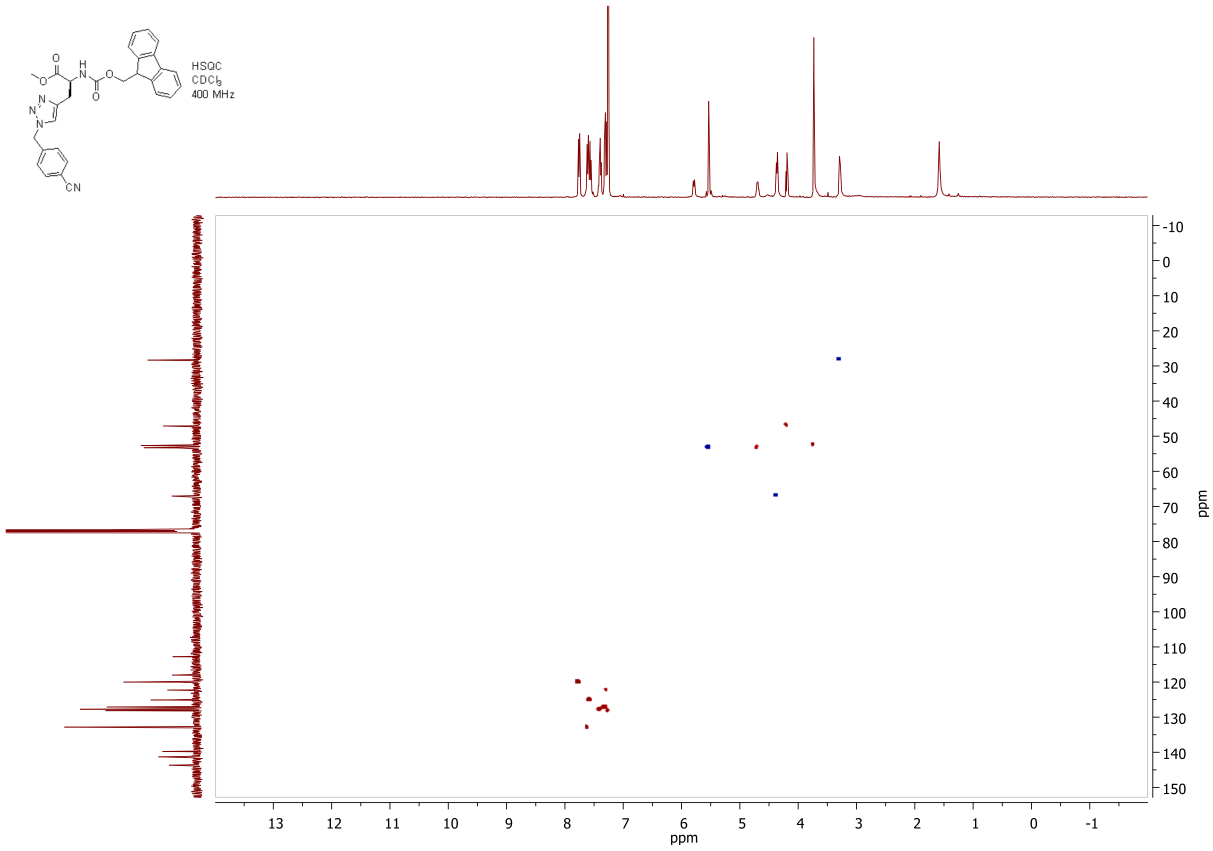
HSQC
CDCl3
400 MHz
ppm
ppm

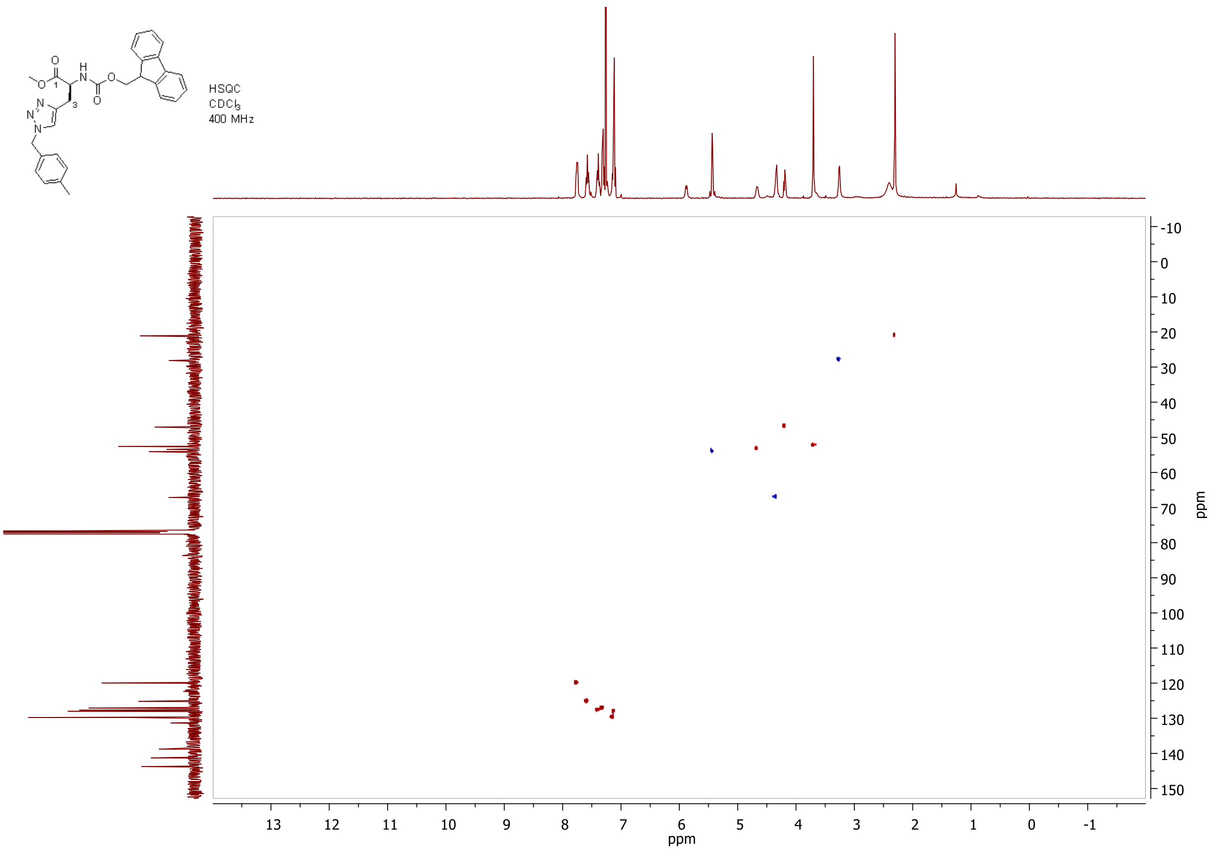
HSQC
CDCl3
400 MHz
ppm
ppm

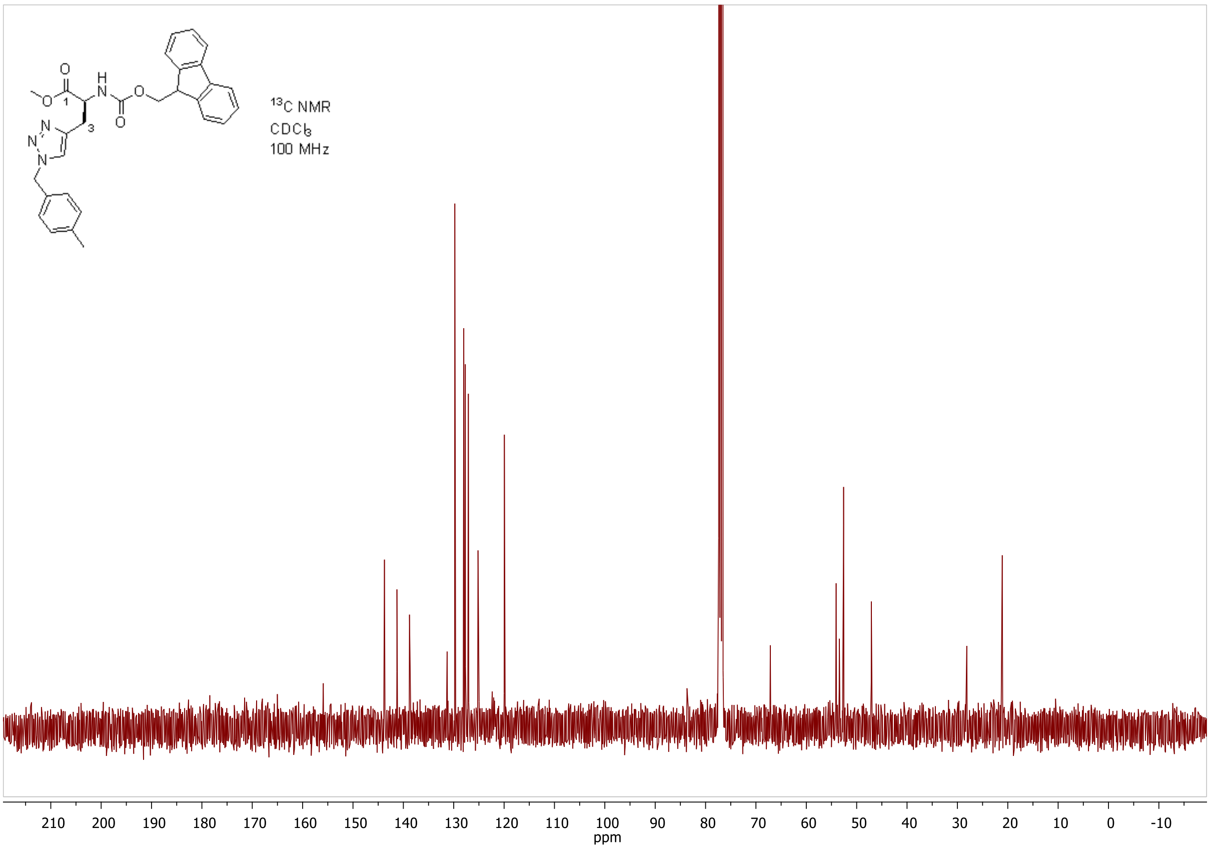
13C NMR
CDCl3
100 MHz
ppm

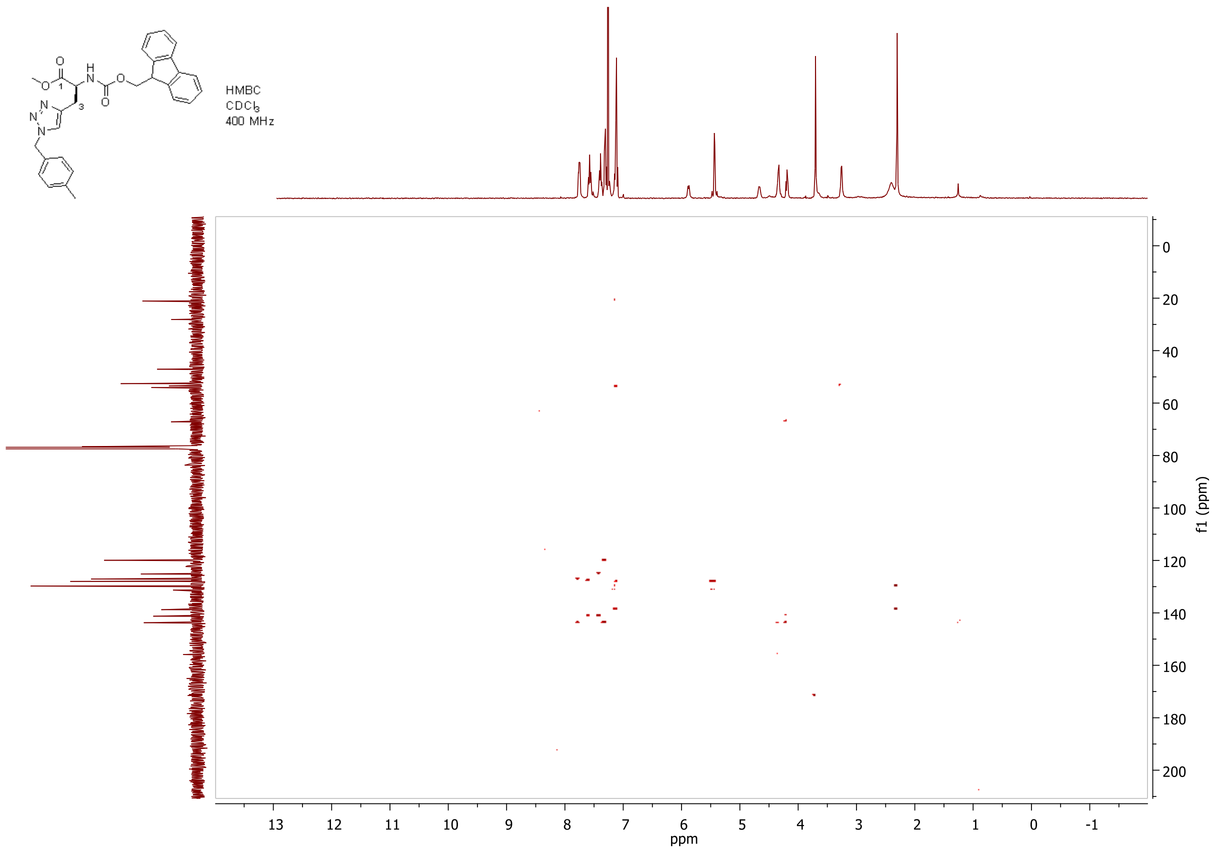
HMBC
CDCl3
400 MHz
ppm
f1 (ppm)

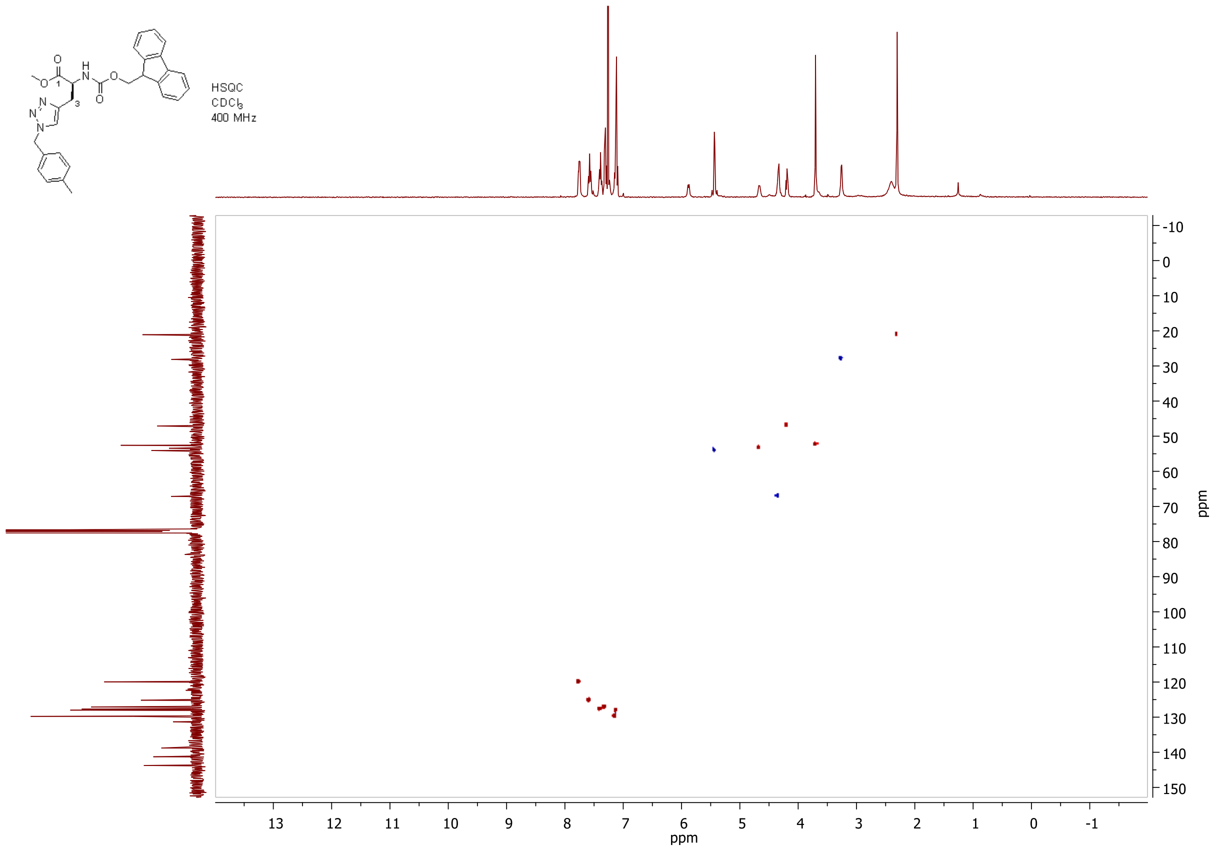
HSQC
CDCl3
400 MHz
ppm
ppm

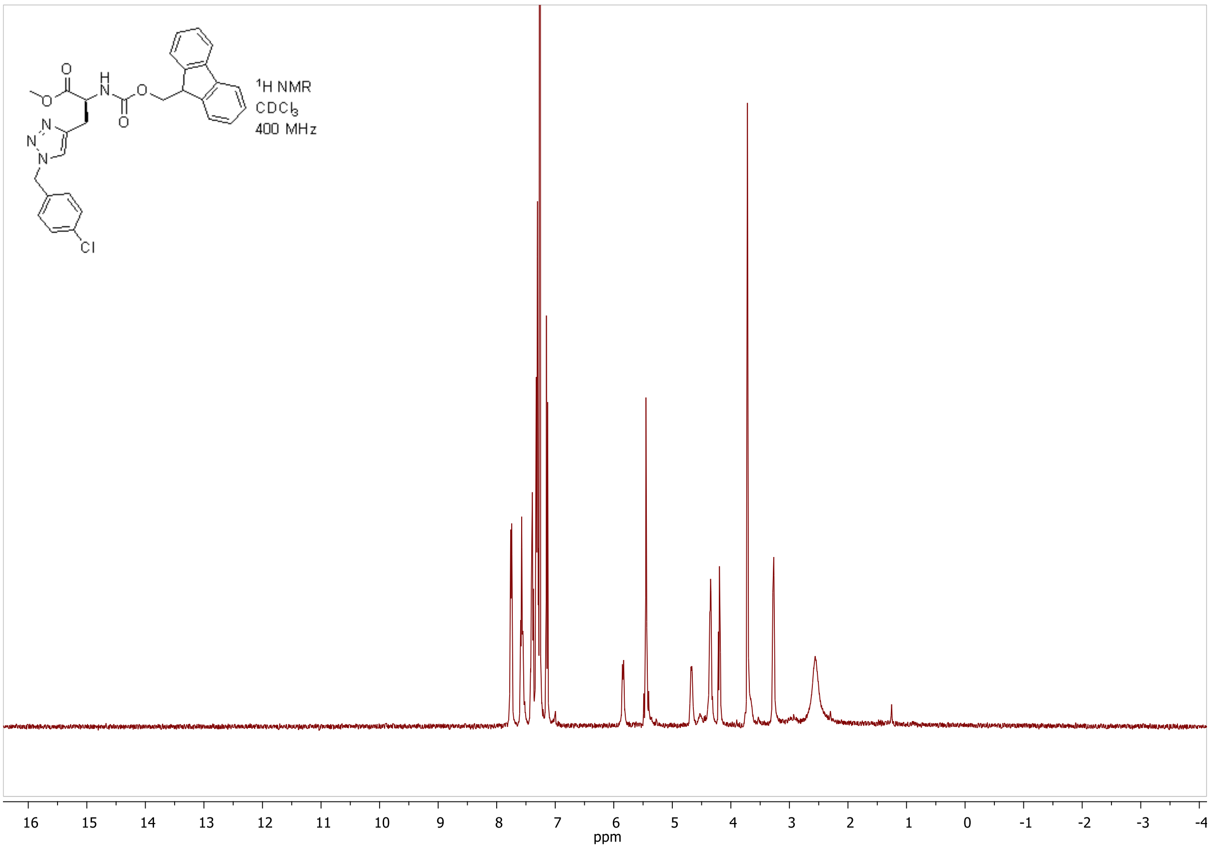
O
O
H
N
O
O
N
N
N
Cl
1H NMR
CDCl3
400 MHz
16
15
14
13
12
11
10
9
8
7
6
5
4
3
2
1
0
-1
-2
-3
-4
ppm

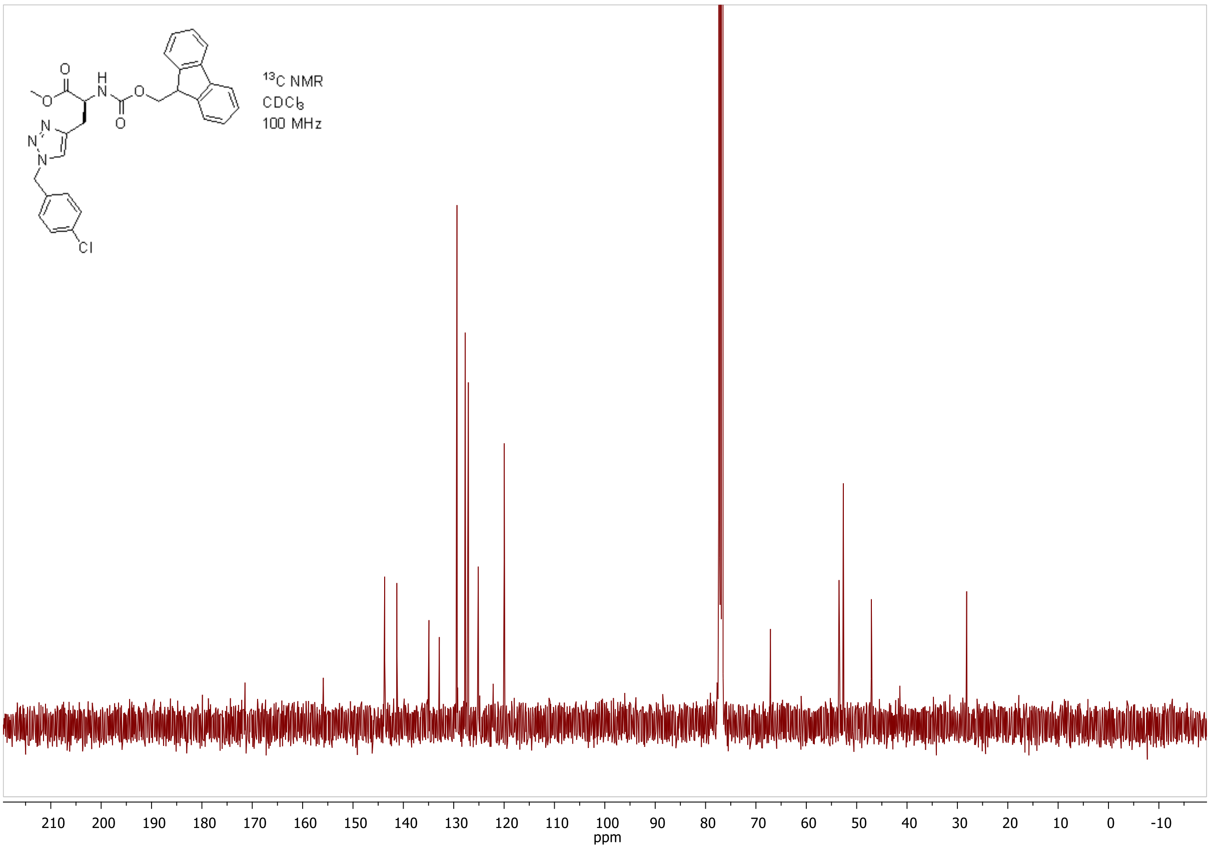
O
O
H
N
O
O
N
N
N
Cl
13C NMR
CDCl3
100 MHz
210
200
190
180
170
160
150
140
130
120
110
100
90
80
70
60
50
40
30
20
10
0
-10
ppm

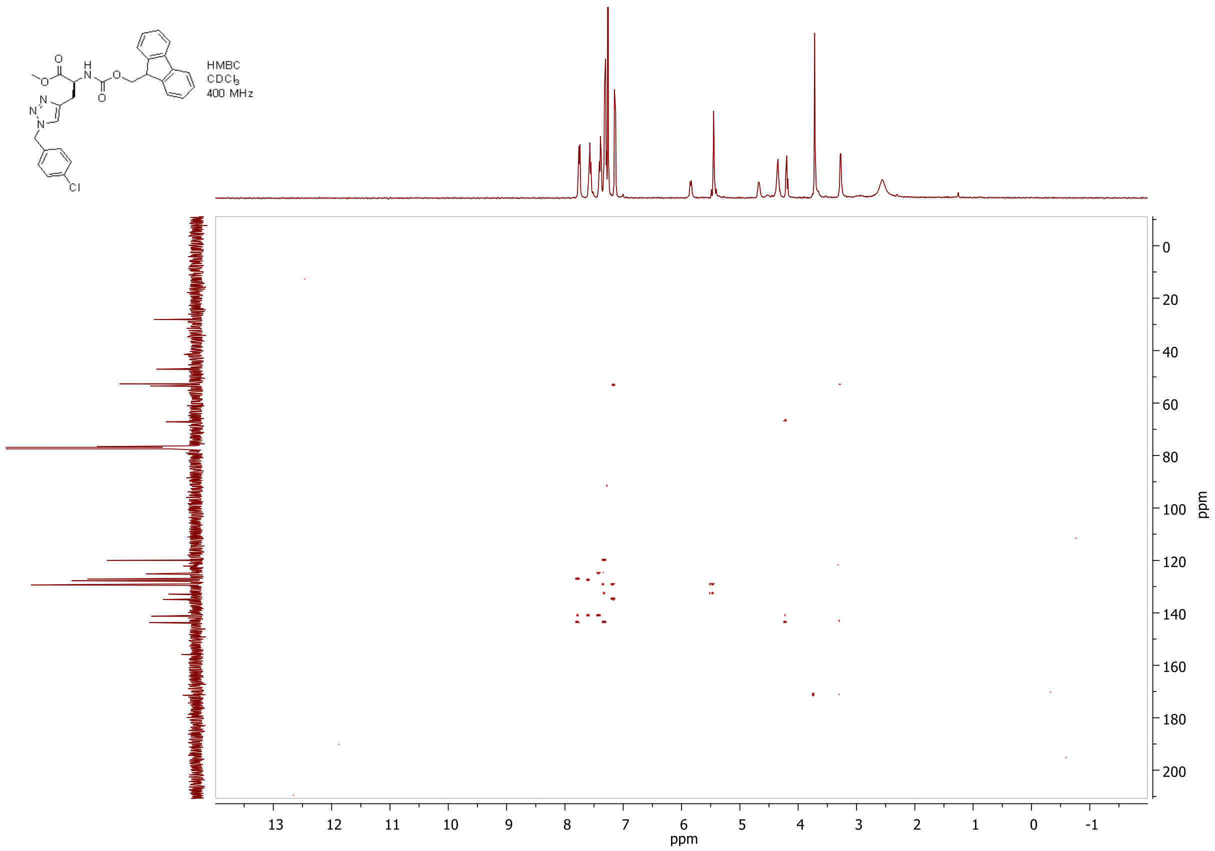
HMBC
CDCl3
400 MHz
ppm
ppm

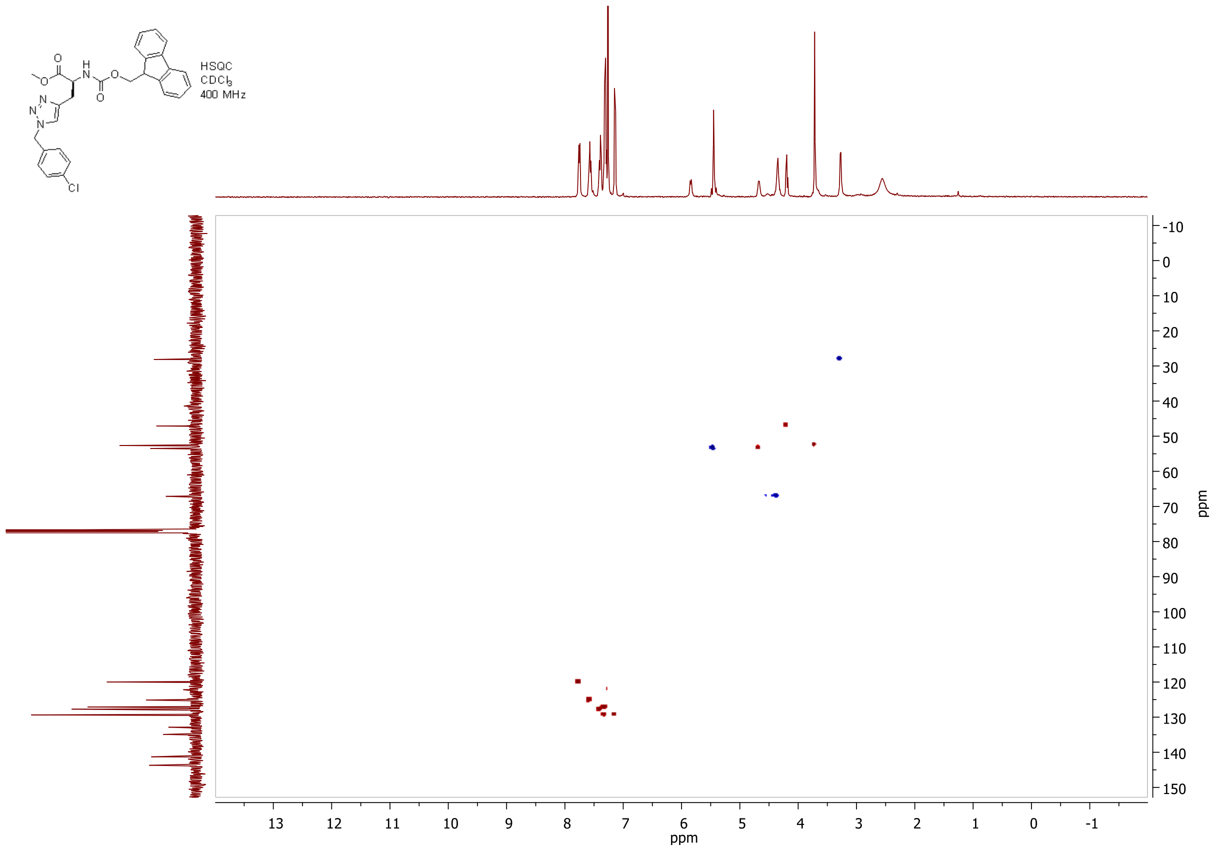
HSQC
CDCl3
400 MHz
ppm
ppm

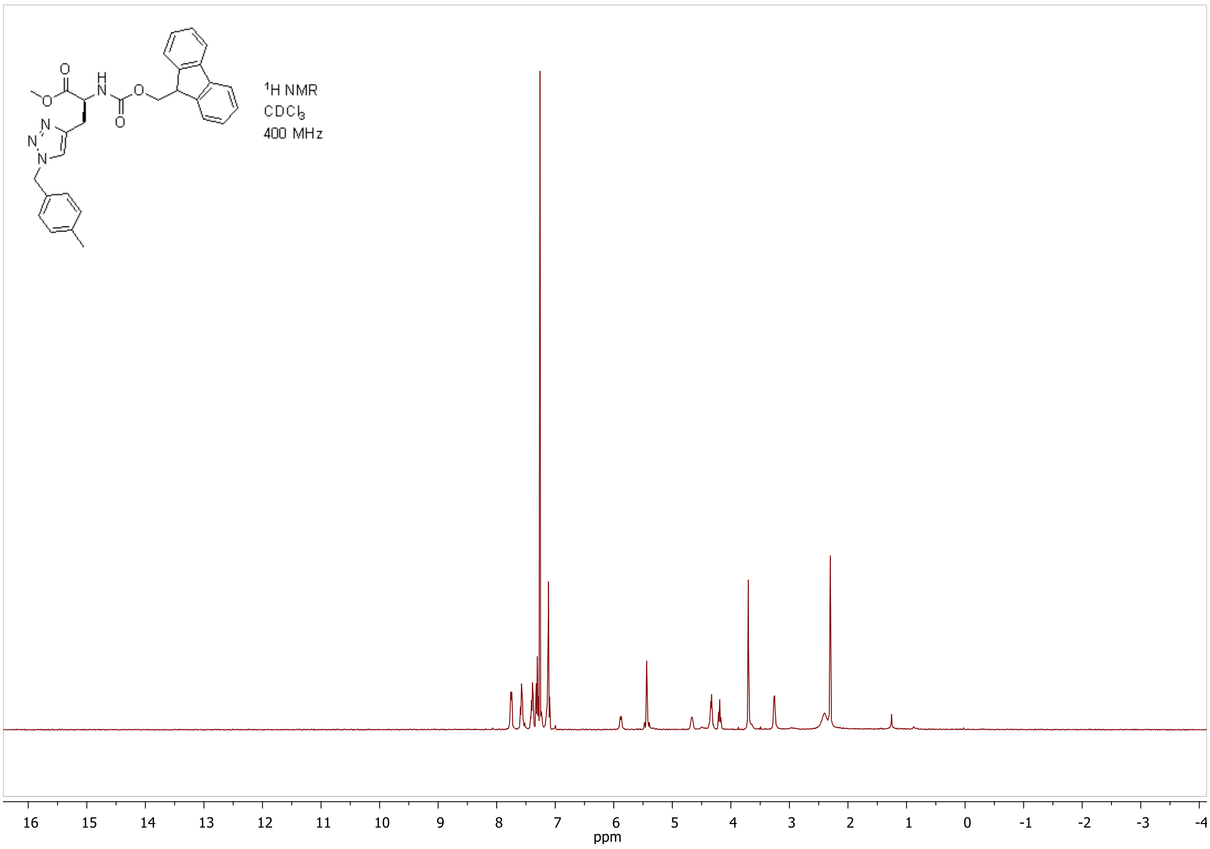
¹H NMR
CDCl3
400 MHz
16 15 14 13 12 11 10 9 8 7 6 5 4 3 2 1 0 -1 -2 -3 -4
ppm

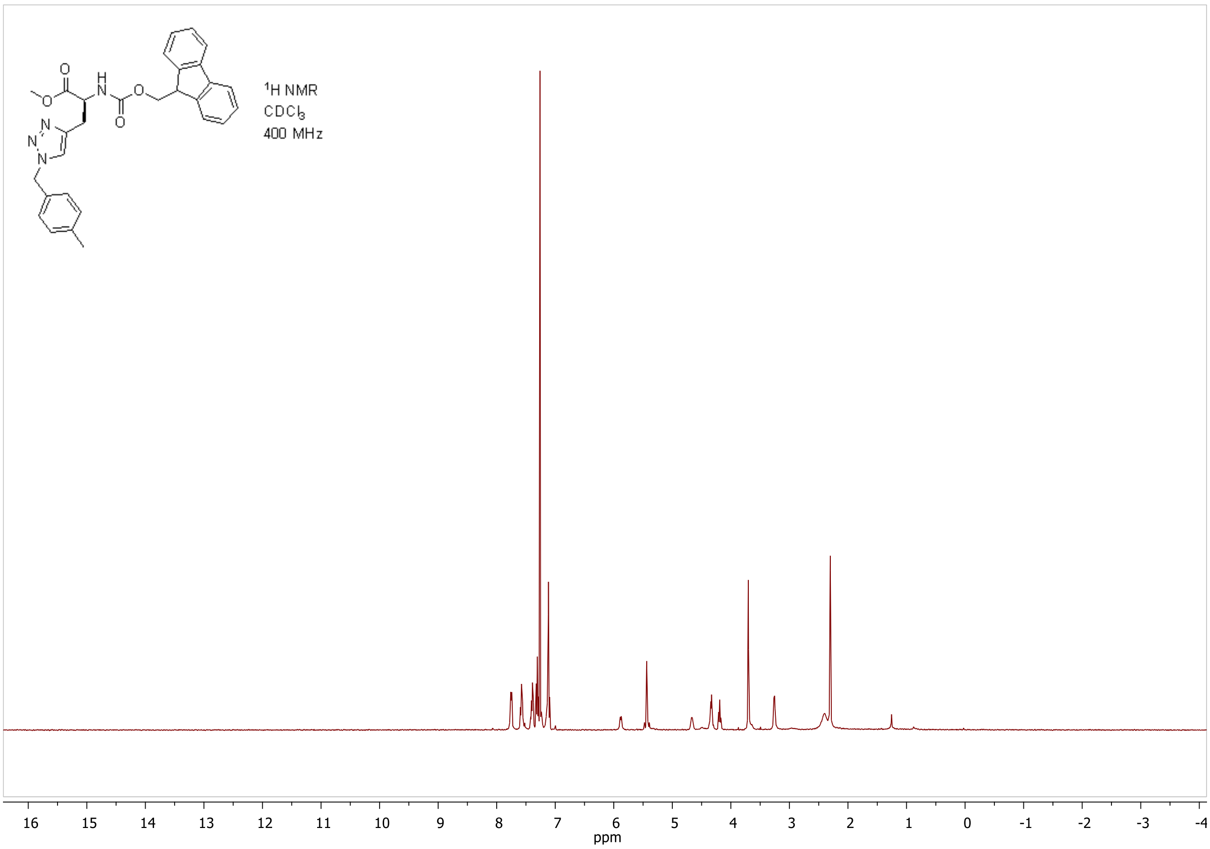
¹H NMR
CDCl3
400 MHz
16 15 14 13 12 11 10 9 8 7 6 5 4 3 2 1 0 -1 -2 -3 -4
ppm

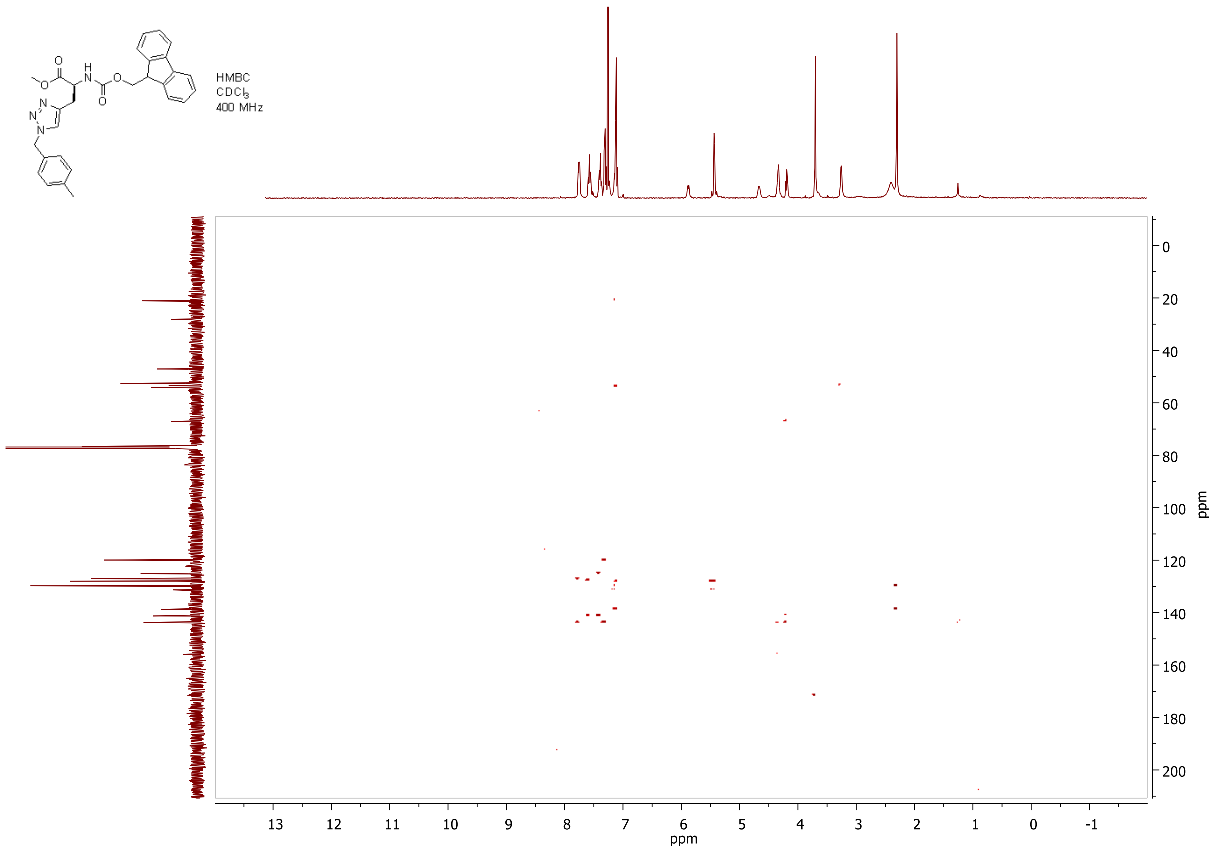

HMBC
CDCl3
400 MHz
ppm
ppm

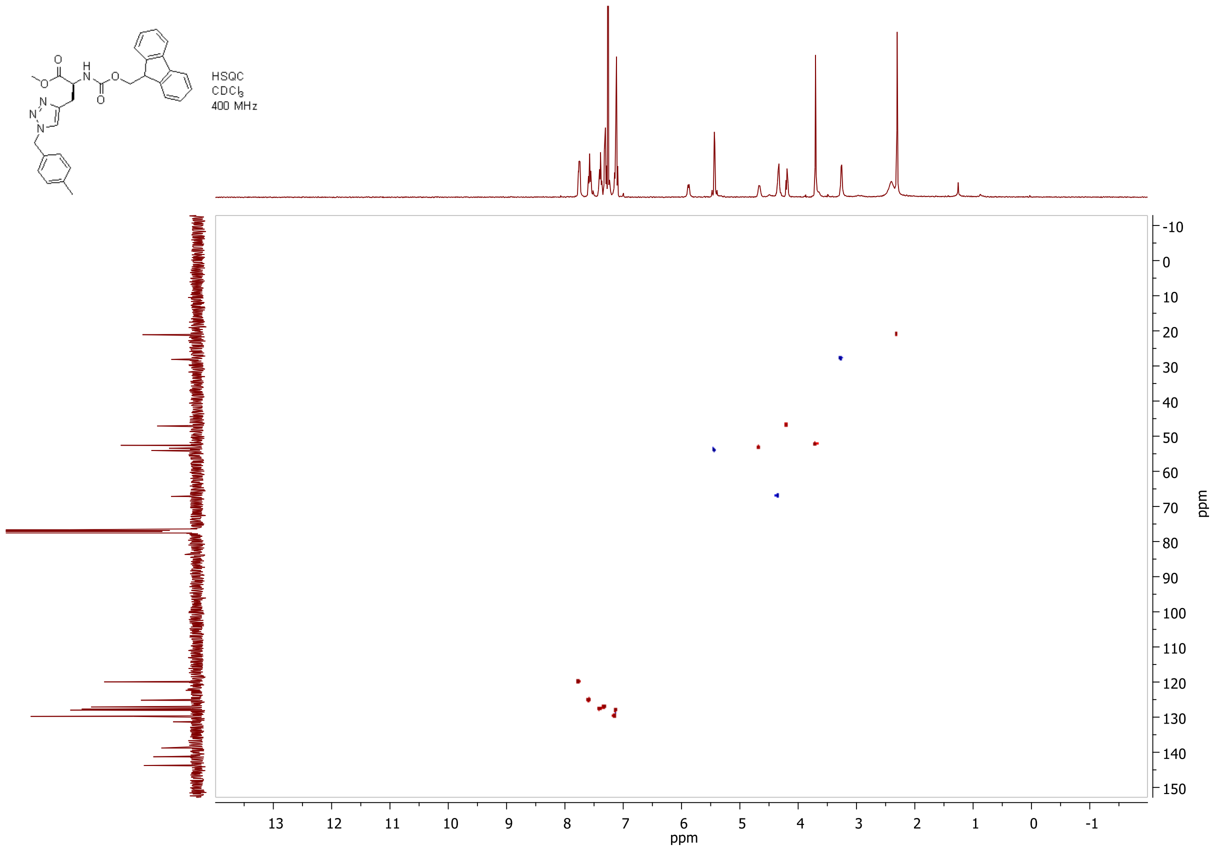

HSQC
CDCl3
400 MHz
ppm
ppm

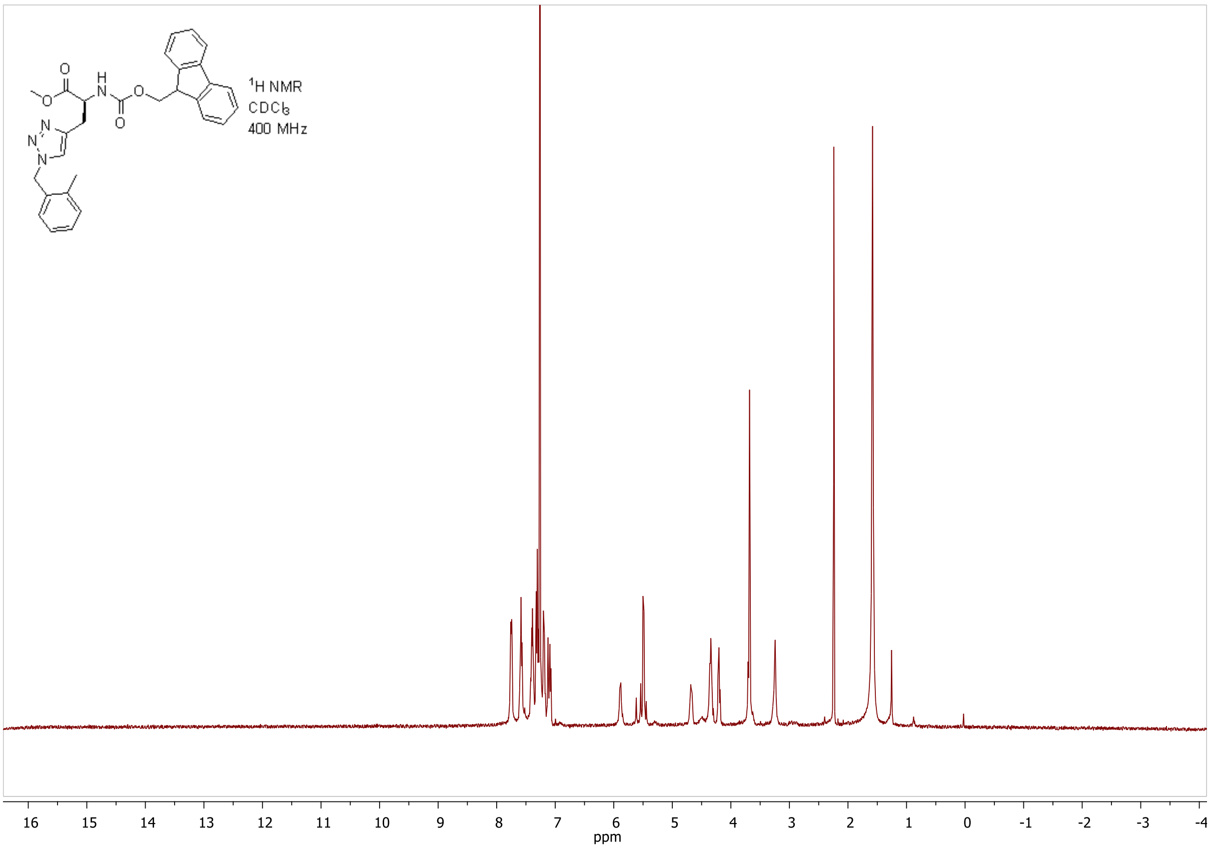

H
N
O
O
O
O
N
N
N
^{1}H NMR
CDCl3
400 MHz
16
15
14
13
12
11
10
9
8
7
6
5
4
3
2
1
0
-1
-2
-3
-4
ppm

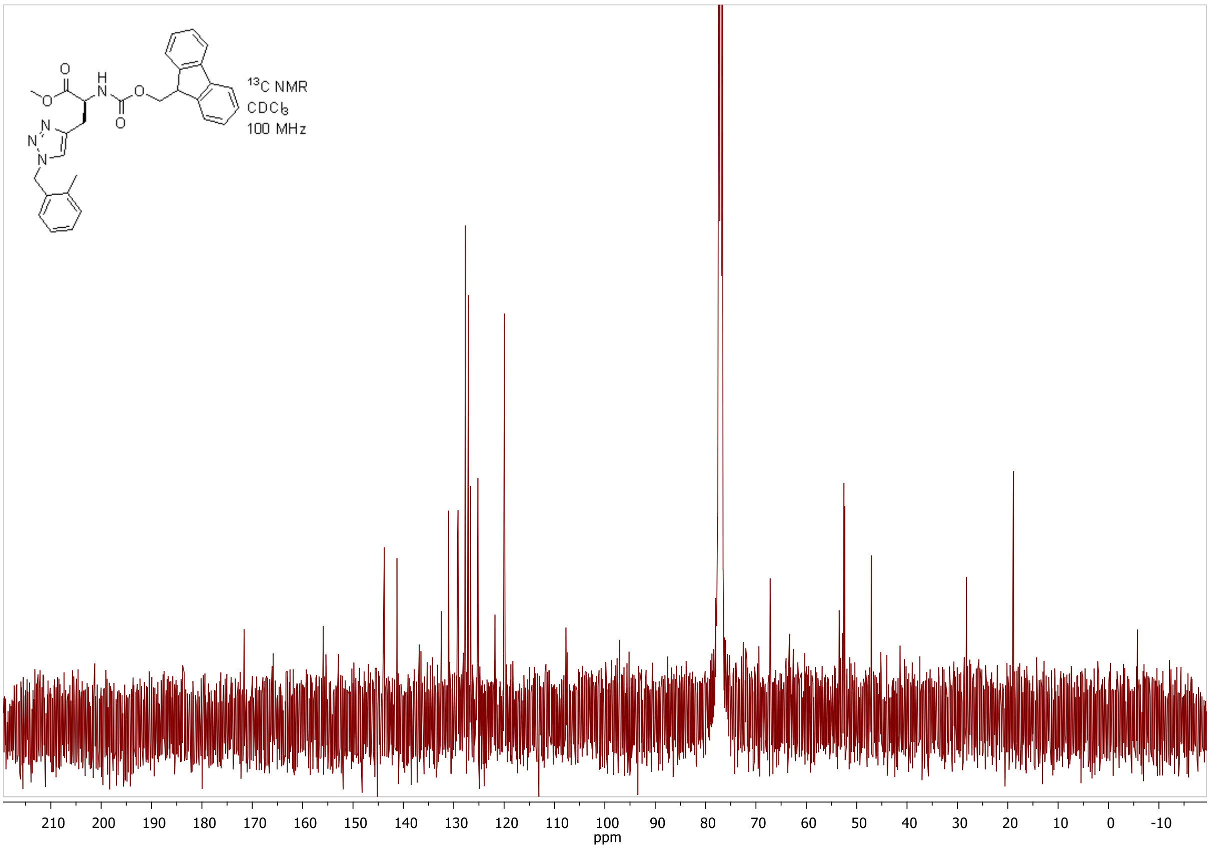

H
N
O
O
O
O
N
N
N
^{13}C NMR
CDCl3
100 MHz
210
200
190
180
170
160
150
140
130
120
110
100
90
80
70
60
50
40
30
20
10
0
-10
ppm

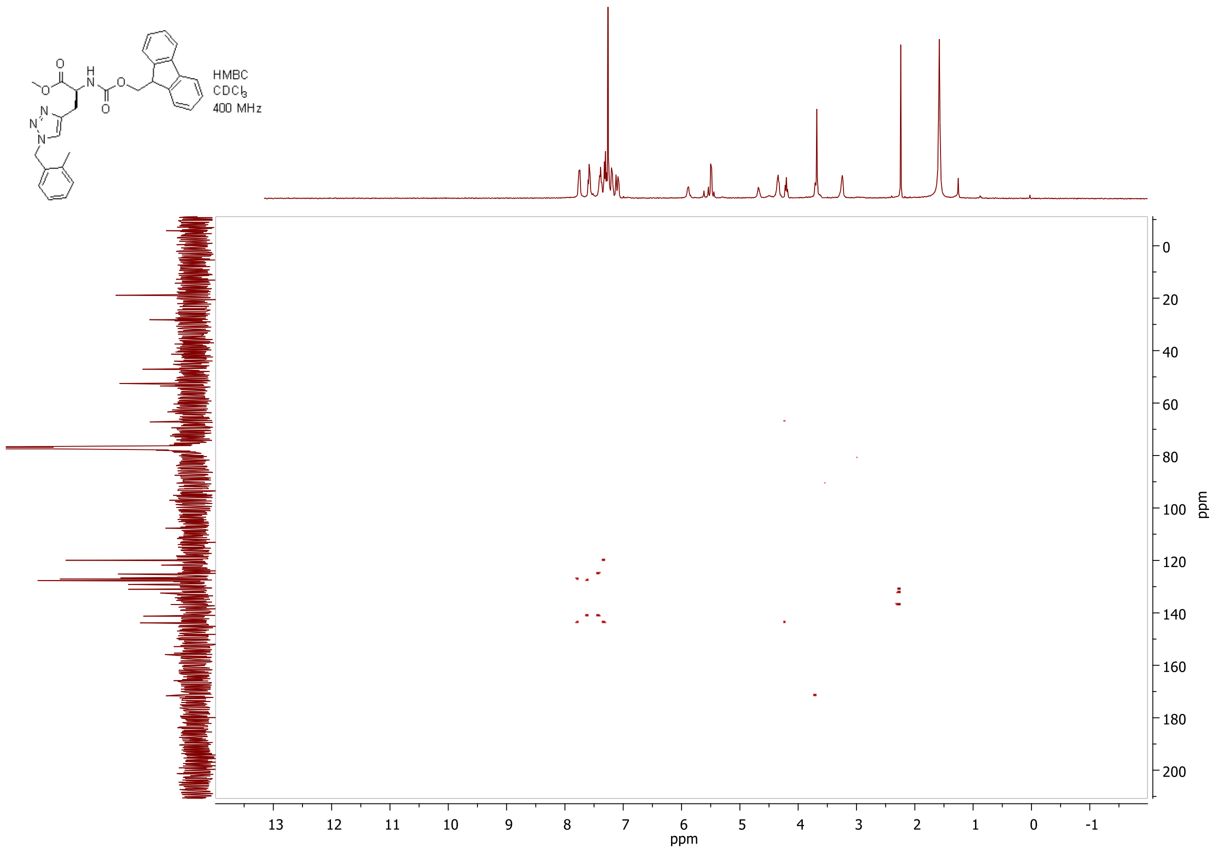
HMBC
$CDCl_3$
400 MHz
ppm
ppm

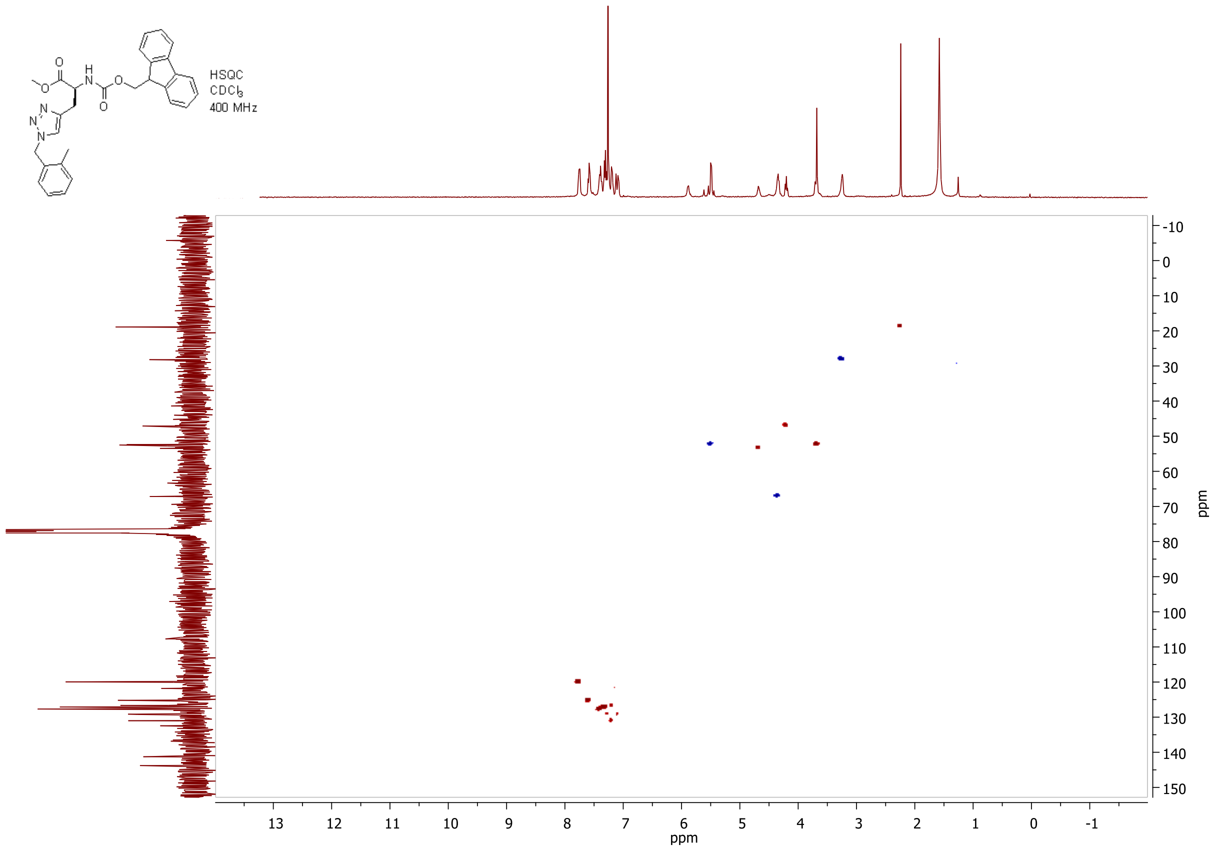
HSQC
$CDCl_3$
400 MHz
ppm
ppm

9. Mcl-1 Fluorescence Anisotropy Assay

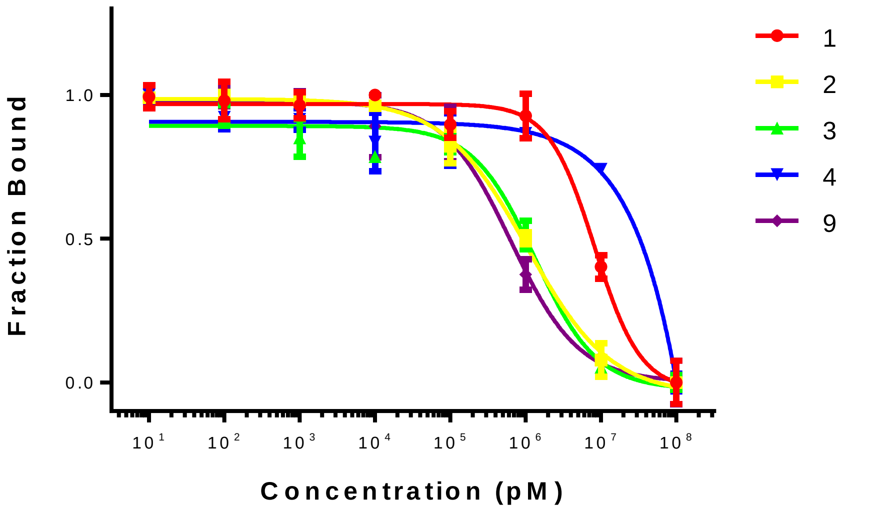

Figure S 6: Fluorescence Anisotropy dose response titration of compounds 1-4 & 9 vs FITC-NoxaB on Mcl-1. Normalized

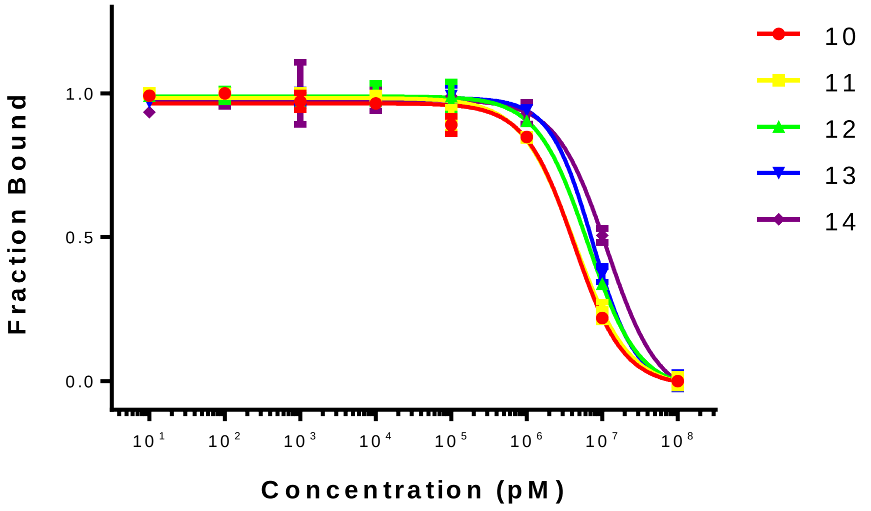

Figure S 7: Fluorescence Anisotropy dose response titration of compounds 10-14 vs FITC-NoxaB on Mcl-1. Normalized

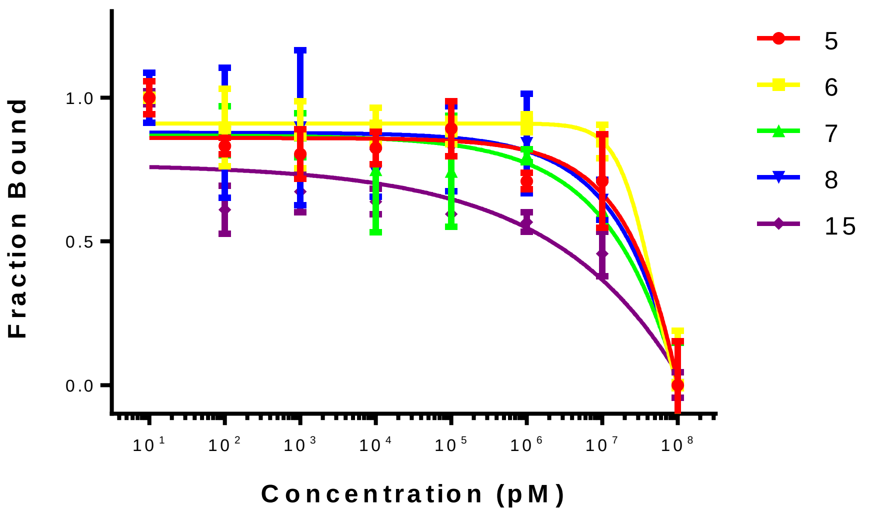

Figure S 8: Fluorescence Anisotropy dose response titration of compounds 5-8 & 15 vs FITC-NoxaB on Mcl-1. Normalized

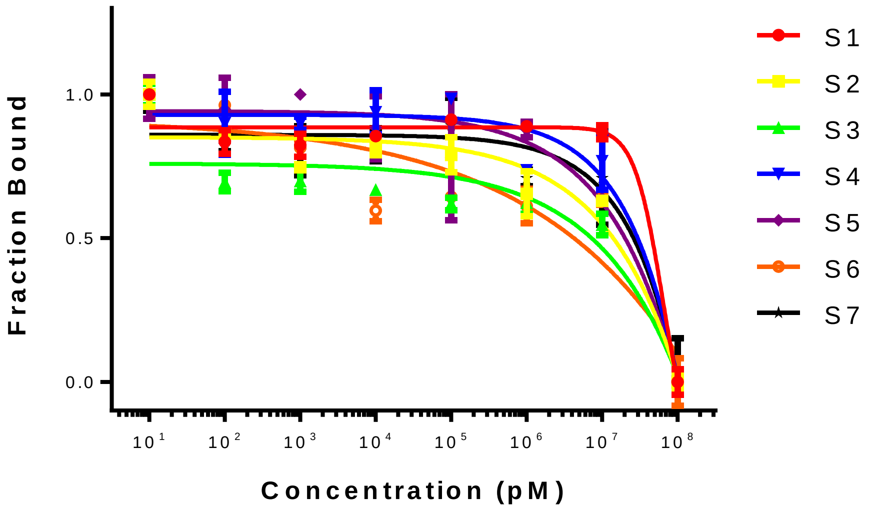

Figure S 9: Fluorescence Anisotropy dose response titration of compounds S1-S7 vs FITC-NoxaB on Mcl-1. Normalized

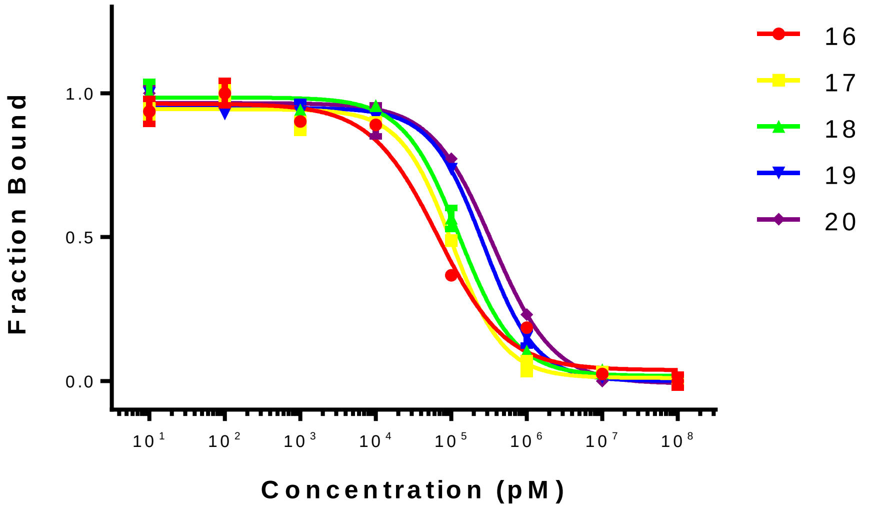

Figure S 10: Fluorescence Anisotropy dose response titration of compounds 16-20 vs FITC-NoxaB on Mcl-1. Normalized

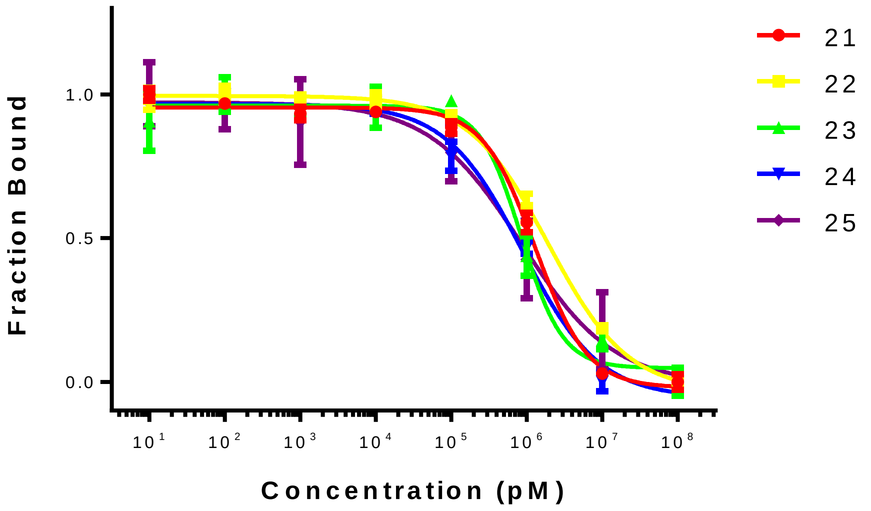

Figure S 11: Fluorescence Anisotropy dose response titration of compounds 21-25 vs FITC-NoxaB on Mcl-1. Normalized

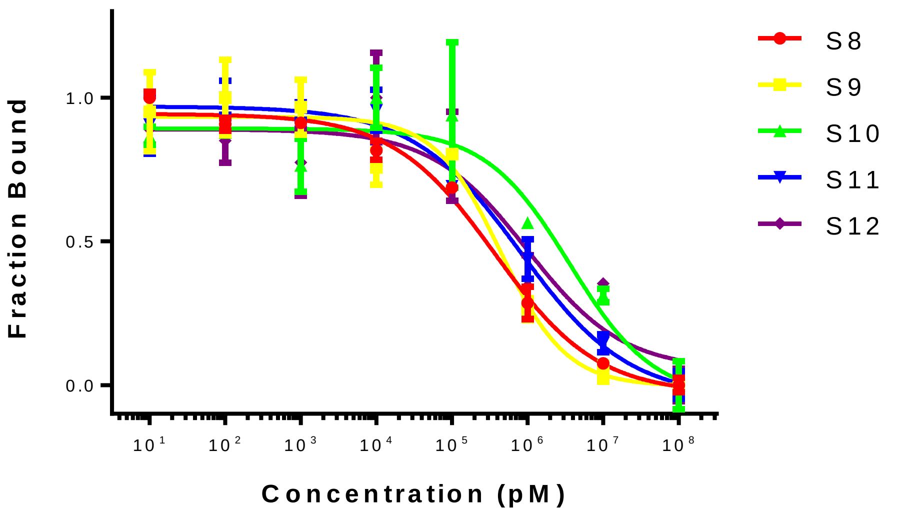

Figure S 12: Fluorescence Anisotropy dose response titration of compounds S8-S12 vs FITC-NoxaB on Mcl-1. Normalized

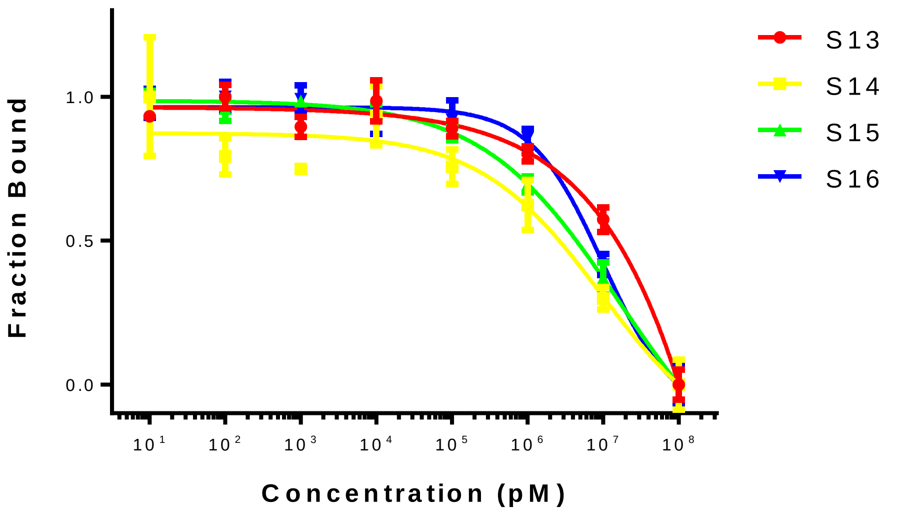

Figure S 13: Fluorescence Anisotropy dose response titration of compounds S13-S16 vs FITC-NoxaB on Mcl-1. Normalized

10. Bcl-2 and Bcl-xL selectivity Fluorescence Anisotropy Assay

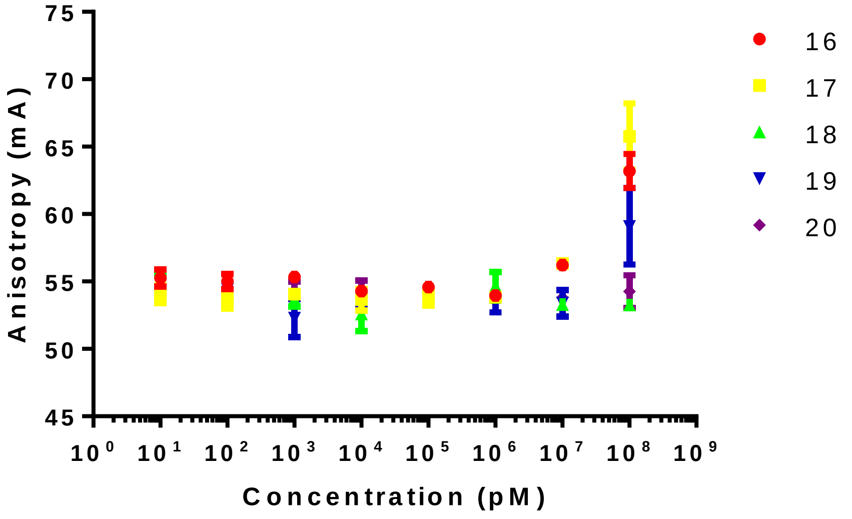

Figure S 14: Fluorescence Anisotropy dose response titration of compounds 16-20 vs FITC-Bid on Bcl-2

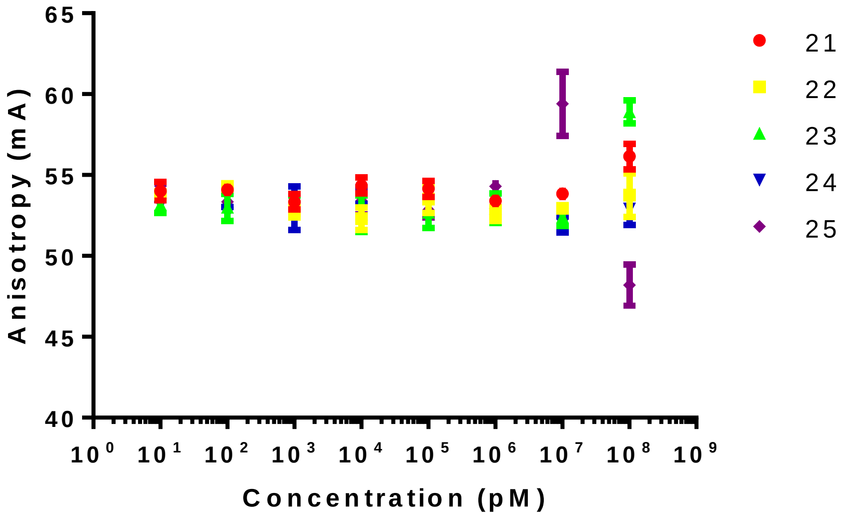

Figure S 15: Fluorescence Anisotropy dose response titration of compounds 21-25 vs FITC-Bid on Bcl-2

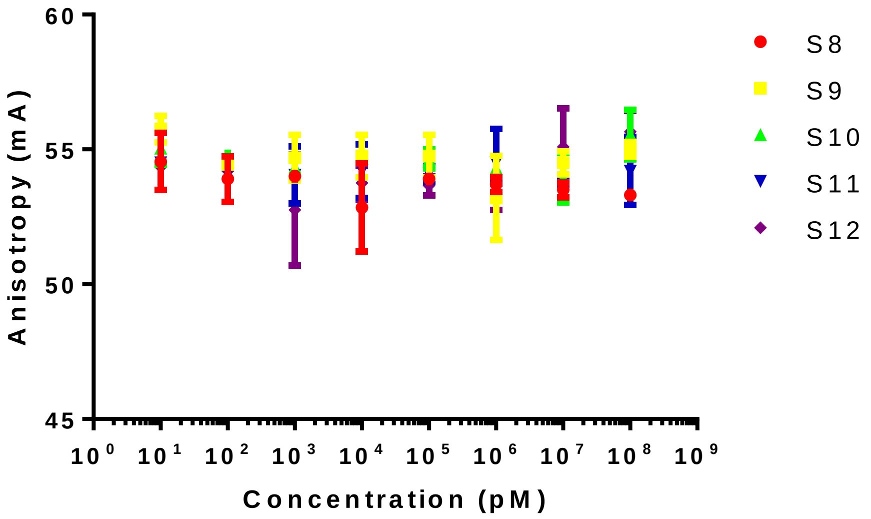

Figure S 16: Fluorescence Anisotropy dose response titration of compounds S8-S12 vs FITC-Bid on Bcl-2

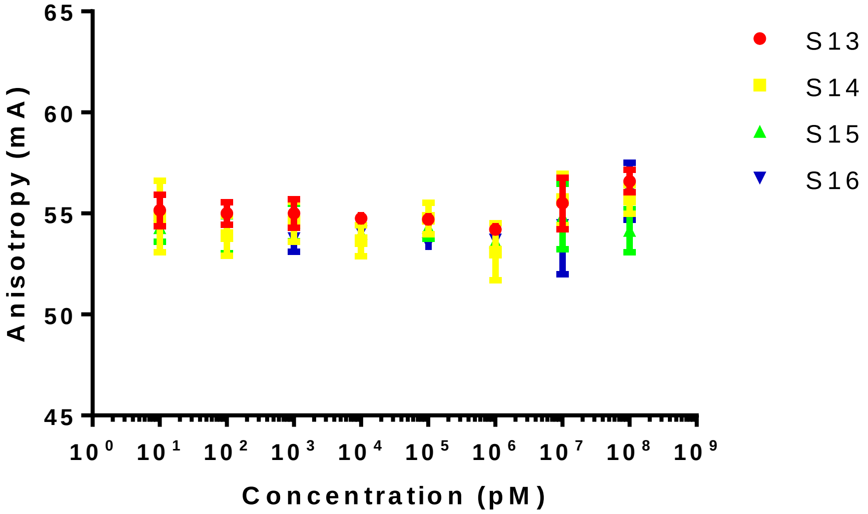

Figure S 17: Fluorescence Anisotropy dose response titration of compounds S13-S16 vs FITC-Bid on Bcl-2

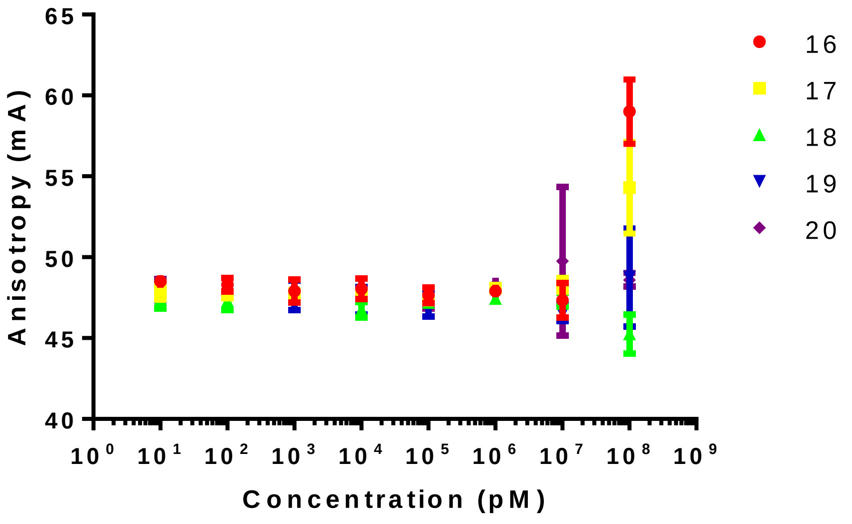

Figure S 18: Fluorescence Anisotropy dose response titration of compounds 16-20 vs FITC-Bid on Bcl-x_L.

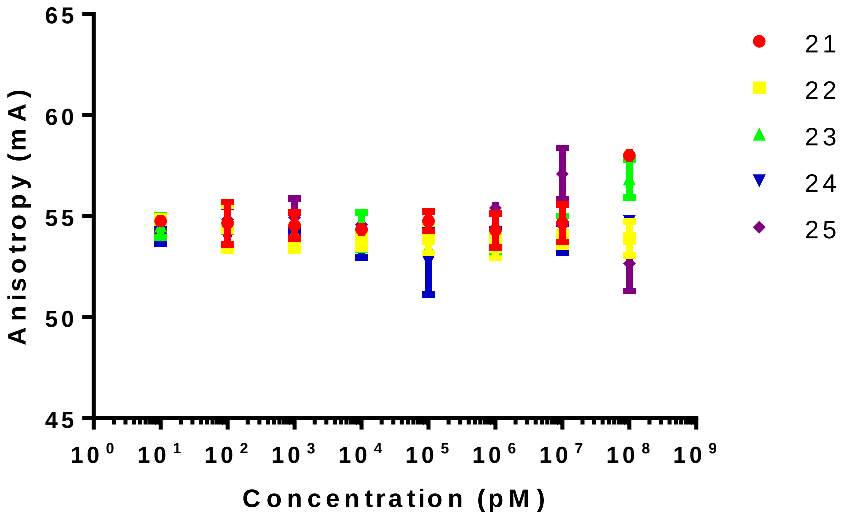

Figure S 19: Fluorescence Anisotropy dose response titration of compounds 21-25 vs FITC-Bid on Bcl-x_L.

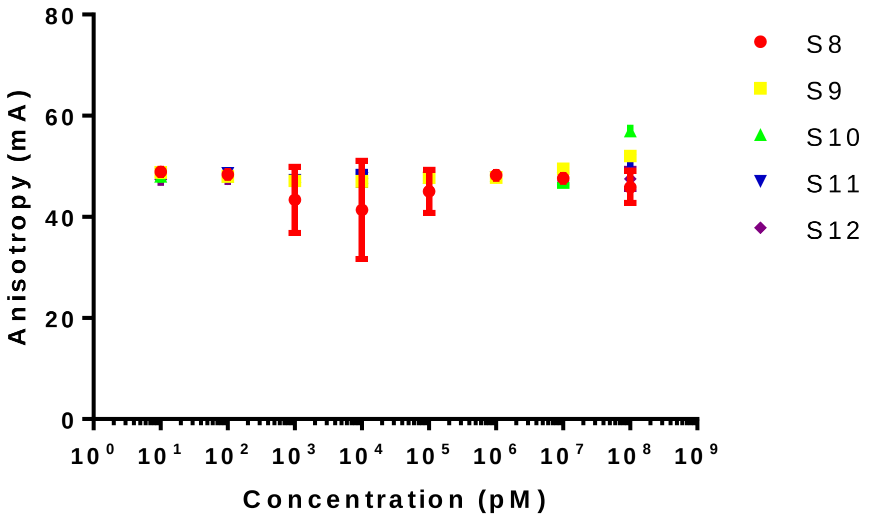

Figure S 20: Fluorescence Anisotropy dose response titration of compounds S8-S12 vs FITC-Bid on Bcl-x_L.

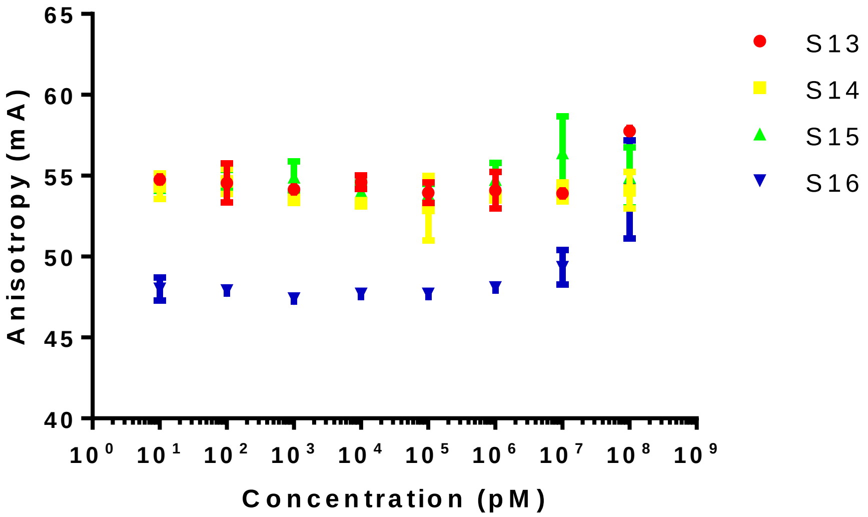

Figure S 21: Fluorescence Anisotropy dose response titration of compounds S13-S16 vs FITC-Bid on Bcl-x_L.

11. Cell growth inhibition assay

MiaPaCa-2

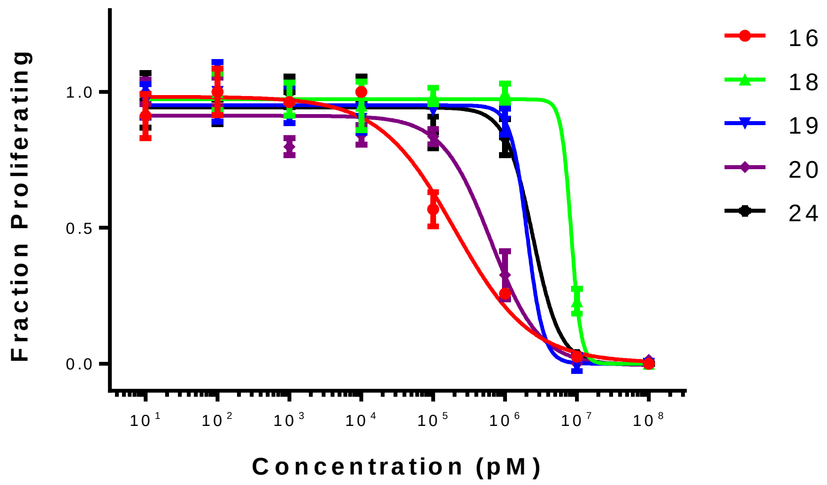

Figure S 22: MTS assay dose response of compounds 16, 18, 19, 20 & 24 towards MiaPaCa-2 cells. Normalized

BxPC-3

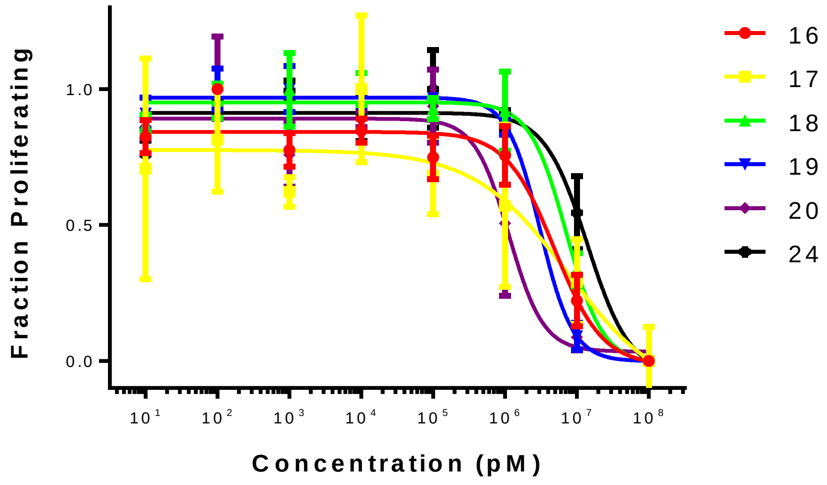

Figure S 23: MTS assay dose response of compounds 16 - 20 & 24 towards BxPC-3 cells. Normalized

ASPC-1

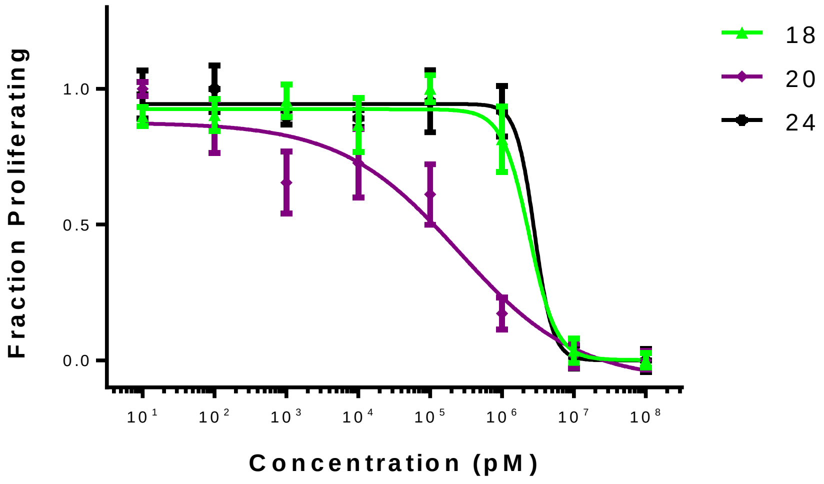

Figure S 24: MTS assay dose response of compounds 18, 20 & 24 towards ASPC-1 cells. Normalized

12. References

[1] B. Zhou, et al., *ChemMedChem* **2011**, *6*, 904-921.
[2] A. M. Petros, et al., *Proc. Nat. Acad. Sci.* **2001**, *98*, 3012-3017.
[3] N. Crawford, et al., *Apoptosis* **2011**, *16*, 311-320.
[4] a) S. Müller, B. Liepold, G. J. Roth, H. J. Bestmann, *Synlett* **1996**, *1996*, 521-522; b) M. Presset, D. Mailhol, Y. Coquerel, J. Rodriguez, *Synthesis* **2011**, *2011*, 2549-2552.
[5] a) P. Thiel, et al., *Chem. Comm.* **2013**, *49*, 8468-8470; b) L.-G. Milroy, et al., *Angew. Chem. Int. Ed.* **2015**, *54*, 15720-15724.
[6] P. E. Czabotar, et al., *Proc. Nat. Acad. Sci.* **2007**, *104*, 6217-6222.
[7] G. M. Sastry, et al., *J. Comput.-Aided Mol. Des.* **2013**, *27*, 221-234.
[8] a) K. Zhu, et al., *J. Chem. Inform. Model.* **2014**, *54*, 1932-1940; b) D. Toledo Warshaviak, et al., *J. Chem. Inform. Model.* **2014**, *54*, 1941-1950.
[9] W. Sherman, H. S. Beard, R. Farid, *Chem. Biol. Drug Design* **2006**, *67*, 83-84.
[10] R. A. Friesner, et al., *J. Med. Chem.* **2006**, *49*, 6177-6196.
